# Ketogenic Diet as an Adjuvant in Epithelial Cancers: Mechanisms, Model Systems, and Translational Opportunities

**DOI:** 10.3390/cancers18162600

**Published:** 2026-08-12

**Authors:** Timo Ylikomi, Matthias Nees

**Affiliations:** 1 Faculty of Medicine and Health Technology, Tampere University, Kalevantie 4, 33100 Tampere, Finland; timo.ylikomi@tuni.fi; 2 Department of Biochemistry and Molecular Biology, Medical University of Lublin, ul. Witolda Chodźki St. 1, 20-093 Lublin, Poland

**Keywords:** ketogenic diet, ketogenic metabolic therapy, epithelial cancer, adjuvant therapy, patient stratification

## Abstract

Ketogenic metabolic therapy (KMT), typically implemented as a ketogenic diet (KD), has re-emerged as a potential adjuvant strategy in oncology. Its rationale is grounded in the metabolic reprogramming of cancer cells, including their reliance on glucose and lipid substrates and the signaling functions of ketone bodies. Despite extensive preclinical evidence of anti-proliferative and immunomodulatory effects, clinical translation in cancer therapy has been slow and inconsistent. This review critically examines the mechanistic basis, experimental and clinical evidence, and translational challenges of KMT across epithelial cancers. We emphasize that “ketogenic interventions” comprise mechanistically distinct modalities, including classical KD, fasting, fasting-mimicking diets, and exogenous ketone supplementation, which are frequently conflated yet differ substantially in their endocrine and metabolic contexts. Across clinical studies, KMT is generally feasible and associated with improvements in quality of life and metabolic parameters; however, robust evidence for the survival benefit of KMT remains limited and heterogeneous. Mechanistically, ketone bodies exert both metabolic and signaling functions, including inhibition of histone deacetylases, lysine β-hydroxybutyrylation, and receptor-mediated effects. Tumor responses are highly context-dependent: ketolytic capacity, governed by enzymes such as OXCT1, can render ketones either a metabolic vulnerability or an alternative fuel for cancer cells. KMT can modify the complex tumor microenvironment, exerting potentially opposing effects on cancer cells, immune cell populations, fibroblasts, and adipocytes. We argue that the central limitation of the field is not a lack of biological activity, but of insufficient integration of tumor-intrinsic metabolism, host physiology, and microenvironmental context. KD/KMT should therefore be regarded not as a universal anticancer therapy, but as a stratified metabolic intervention, whose progress will depend on biomarker-guided patient selection, improved experimental models, and rational combination strategies within a “press-pulse” therapeutic framework.

## Simple Summary

Cancer cells often rely heavily on glucose to fuel their growth. The ketogenic diet, which is very low in carbohydrate and high in fat, lowers blood sugar and raises molecules called ketone bodies; it has been proposed to make tumors more vulnerable while supporting patient well-being. In this review, we bring together laboratory findings and results from patient studies across common epithelial cancers, including breast, prostate, pancreatic, colorectal, and lung cancer. We explain why the diet’s effects vary widely from one tumor or patient to another, and why it generally works best alongside standard treatments rather than on its own. We also highlight which patients are most likely to benefit, which laboratory models best capture the underlying biology, and which simple measurements could guide future use, with the aim of a more precise, individualized metabolic approach.

## 1. Ketogenic Diet (KD) and Ketogenic Metabolic Therapy (KMT)

### 1.1. The Unmet Medical Need: Modest Gains in Advanced and Metastatic Cancer

This review focuses primarily on epithelial malignancies, and considers the ketogenic diet (KD) and ketogenic metabolic therapy (KMT) as an adjuvant to standard anticancer treatment, rather than as a replacement for therapies. Glioblastoma is included only as a historically informative comparator for which a large body of evidence is available. Throughout, we treat classical KD, fasting, fasting-mimicking diet (FMD), caloric restriction (CR), and exogenous ketone supplementation as mechanistically distinct interventions that significantly differ in their endocrine and metabolic contexts and should not be conflated.

Two terms recur throughout: (1) The “press-pulse framework” denotes a strategy in which a sustained metabolic modification provides a continuous “press,” while time-limited standard treatments such as chemotherapy or radiotherapy deliver the therapeutic “pulses”—this concept does not imply that KD is independently curative. (2) By monotherapy, we mean the use of KD/KMT without concurrent tumor-directed treatment. The currently available evidence does not support KD or KMT as a replacement for state-of-the art clinical regimen including surgery, radiotherapy, systemic therapy, or immunotherapy.

Despite decades of therapeutic innovation, the prognosis for patients with advanced and especially metastatic cancers remains poor. For localized malignancies, multimodal therapies, including improvements in surgical techniques and chemo- and radiotherapy, have substantially improved patient survival. However, for patients with distant or locally advanced disease, systemic pharmacotherapy continues to offer only a small, incremental benefit, if any, and its impact on patient quality of life is often negative. This reality is illustrated by a series of rigorous analyses of recent approvals of cancer drugs. Across diverse tumor types, the median overall survival (OS) or progression-free survival (PFS) improvement achieved by newly approved cancer drugs is typically only in the range of a few months:

• A recent study in *The Lancet* examined several recent cancer drug approvals by the FDA, and reported an average median overall OS benefit of only 2.4 months for the patients enrolled in these clinical trials (range: 1.25–3.89 months) [1].
• This confirms an earlier study, published by the European Medicines Agency (EMA), that summarized all anticancer drug approvals from 2009 to 2013. This report showed a comparable, generally low median OS gain of just 2.7 months (range: 1.0–5.8 months) [2].
• Another recent analysis of 124 anticancer drugs approved between 2003 and 2021 (published in 2023) found a low median OS prolongation of only 2.8 months and a progression-free survival (PFS) benefit of no more than 3.3 months [3].
• Finally, a systematic review of 319 lung cancer trials from the year 2025 reported a mean OS improvement of only 2.28 months [4].

**Figure 1:**
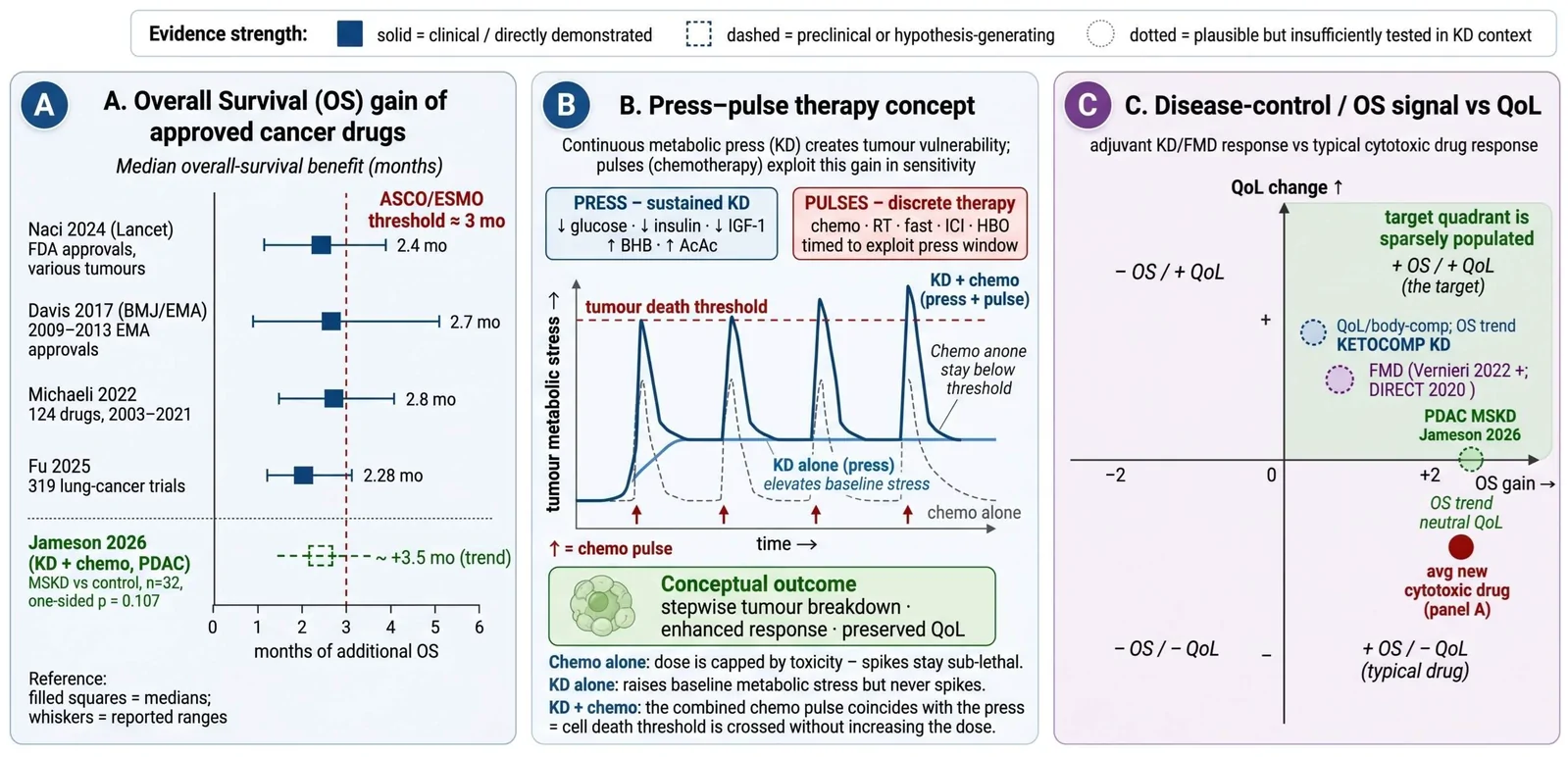
The clinical unmet need and the press-pulse rationale. A three-panel figure: (**A**) median overall-survival gains of recently approved oncology drugs cluster at or below the threshold for clinically meaningful benefit; (**B**) a continuous ketogenic press lowers tumor metabolic resilience so that discrete therapeutic pulses (chemotherapy, radiotherapy, fasting, ICI, hyperbaric oxygen) act more effectively; and (**C**) the rare upper-right quadrant of positive OS combined with positive QoL is the therapeutic target.

This concerning picture (summarized in Figure 1A,C) is even more limited when the overall improvement in the patient Quality of Life (QoL) is considered. Only 10% of EMA-approved cancer drugs demonstrated any significant QoL improvement at the time of market authorization [2,5,6]. Among 45 phase 3 trials conducted in 2019, only 24% showed significant improvement in global QoL [5]. Critically, no statistically significant association was found between progression-free survival benefit and QoL improvement [5]. As we discuss throughout this review, even modest improvements in survival or disease control that are simultaneously accompanied by significant, measurable QoL benefits represent a qualitatively distinct and clinically meaningful form of therapeutic gain, particularly when these therapies are benchmarked against the prevailing standard. Ketogenic metabolic therapy (KMT), or the “ketogenic diet” (KD), as we will call it here most of the time, has been proposed as one candidate to occupy this position. This applies especially when KMT/KD is used as an adjuvant combined with established standard-of-care clinical treatments. This is also closely related to fasting-mimicking diet (FMD), as pioneered by Valter Longo [7,8,9].

### 1.2. Ketogenic Metabolic Therapy/Ketogenic Diet: From Monotherapy to Rational Adjuvant

KD represents a high-fat, very low-carbohydrate diet, or “nutritional intervention” that can potently shift systemic metabolism toward ketone body production and utilization. KD has been tested and explored specifically in oncology, originally based on the early hypothesis that restricting glucose availability might selectively disadvantage cancer cells while sustaining overall normal tissue function [10,11]. The mechanistic rationale is grounded in the widespread metabolic reprogramming of tumor cells: many cancers exhibit a high dependence on glucose, driven by the Warburg effect [12,13,14] and oncogenic rewiring of glycolytic and anabolic pathways. Essentially, the Warburg effect implies that tumor cells produce ATP via glycolysis at a rate faster than via respiration [13]. Later, we will also describe the possibility of a reverse Warburg effect [15], which essentially relates to effective aerobic glycolysis by cancer-associated fibroblasts (CAFs) and the tumor stroma [16], and not the tumor cells themselves.

By reducing circulating glucose and insulin levels and simultaneously elevating β-hydroxybutyrate (BHB) [17] and acetoacetate (AcAc) levels, the ketogenic metabolic state directly targets this dependency, which represents a therapeutically exploitable metabolic vulnerability in selected tumor contexts. Ketone bodies can also exert direct anti-tumor effects through epigenetic mechanisms, including inhibition of histone deacetylases (HDACs). Ketone bodies further target lysine β-hydroxybutyrylation (Kbhb) of histones, an epigenetic modification that affects the expression of signaling receptors [18,19,20], and modulates genes such as GPR109A and NLRP3 [21]. Histone Kbhb exerts bidirectional regulatory effects on tumor metabolism, cardiovascular diseases, immune regulation, and many other processes. Furthermore, Kbhb targets immunomodulatory activity within the tumor microenvironment (TME), which we will review in detail in Section 3 and Section 4.

A critical insight from a decade of clinical investigation, however, is that ketogenic metabolic therapy (KMT) is unlikely to achieve clinically meaningful anti-tumor effects, especially when employed as a monotherapy. Phase 1 trials in glioblastoma currently represent the most extensively studied tumor type [22,23]. Multiple glioma studies have generally demonstrated the safety and feasibility of KD. However, they have also shown limited survival benefit when used as a standalone intervention [24]. This mirrors a broader pattern observed consistently in cancer therapy: single-agent approaches, including metabolic strategies, are frequently circumvented by the high metabolic plasticity of tumors (and the general plasticity of cancer cell behavior under stress conditions). This can be achieved through substrate switching and is further supported by the intrinsic redundancy and connectivity of most, if not all, energy-generating pathways. The clinical promise of ketogenic metabolic therapy, or KD, lies not in the replacement of established treatments. The available evidence most strongly supports developing KD/KMT as an adjuvant strategy rather than as monotherapy: KD/KMT may metabolically prime tumors and host tissues for improved responses to selected standard therapies, including cytostatic chemotherapy, radiotherapy, and immunotherapy, although this remains largely context-dependent and incompletely validated clinically [25,26,27]. Simultaneously, it appears that KD supports patient well-being during treatment and often significantly improves their QoL.

Supporting this adjuvant framing for KD, recent systematic evidence confirms that ketogenic interventions improve precisely those QoL parameters that established cancer pharmacotherapy and novel, experimental chemotherapies regularly fail to address. A recent meta-analysis of 14 controlled clinical trials that include KD in at least one arm, encompassing over 500 patients [28], reported significant improvements in patient emotional functioning. This includes insomnia, social functioning, and fatigue. In addition, this study observed anti-inflammatory effects, as evidenced by a reduction in C-reactive protein (CRP) levels. These are reminiscent of “anti-inflammatory diets” explored in various other studies [29]. The anti-inflammatory effects of KD were most pronounced in studies lasting more than 12 weeks. This suggests that sustained metabolic modification by KD is required to achieve full therapeutic potential. Their meta-analysis identified optimal macronutrient ratios for patients consisting of 2–4% carbohydrates, 16–18% protein, and 80–85% fat [28].

A conceptual framework that articulates the rationale for adjuvant therapy is the “press-pulse” model of metabolic therapy [30]. This strategy combines a sustained metabolic press (i.e., the chronic reduction of circulating glucose and elevation of ketones through dietary intervention) followed by episodic metabolic pulses. This concept is illustrated in Figure 1B. The pulses are precisely timed to fully exploit the vulnerabilities created by the press phase. The “press” phase can be further complemented, at least in patients for whom this is appropriate, by additional administration of pharmacological agents, such as mechanistic target of rapamycin (mTOR) or phosphatidylinositol 3-kinase (PI3K) inhibitors [31]. Tong et al. showed that liraglutide, a GLP-1 receptor agonist, could effectively block cell cycle progression in colorectal cancer, reduce cell proliferation, migration, and invasion, and also promote apoptosis, essentially by inhibiting the PI3K/Akt/mTOR signal pathway. Rapamycin and clinical mTORC1 inhibitors or “Rapalogs” (such as Everolimus, and Temsirolimus) have strong preclinical support in combination with KD [32]. Furthermore, KD rescues the efficacy of PI3K inhibitors by suppressing the insulin feedback loop, and a similar logic applies to allosteric mTORC1 inhibitors. The “pulses” used in this therapy may include short-term fasting, hyperbaric oxygen, or targeted pro-oxidant treatments, or combinations thereof. Targeted pro-oxidant approaches (e.g., by hyperbaric O2, vitamin C megadoses, and atovaquone [33,34,35]) are becoming an active area within the press-pulse framework and will be discussed in more detail in Section 8. The press-pulse strategies are generally designed to lower the therapeutic threshold for existing anticancer treatments while simultaneously reducing systemic toxicity for patients. The past and present landscape of clinical trials involving either KD or fasting-mimicking diet (FMD) is reviewed in more detail in Section 2 and Section 3 of this review; see Box 1 for comparisons. Consistent with systemic target engagement in humans, a recent presurgical randomized study in endometrial cancer, published in 2026, found that a short very-low-carbohydrate ketogenic intervention markedly reduced circulating insulin and was associated with reduced tumor p-AKT (see Section 2.3). This supports rational evaluation of ketogenic intervention(s) alongside PI3K-pathway inhibitors, although clinical efficacy yet remains to be established [36].

Box 1 **A note on nomenclature and distinct interventions. The general term “ketogenic intervention” is used in the literature to refer to at least four mechanistically non-interchangeable regimens. These distinctions matter for both mechanisms and clinical interpretation:**

(i) A classical ketogenic diet (KD) is a sustained high-fat, low-carbohydrate dietary pattern producing mild nutritional ketosis (BHB 0.5–3 mM); caloric intake is typically maintained [22,37,38]. An effective KD was identified as an optimal macronutrient ratio for patients, consisting of only 2–4% carbohydrates, 16–18% protein, and 80–85% fat (expressed as % of total energy intake). It is often termed synonymously ketogenic metabolic therapy (KMT).

(ii) Prolonged fasting produces deeper ketosis (BHB 2–8 mM) combined with negative energy balance, protein catabolism, and a strong decline in the levels of insulin, IGF-1, and glucose. These effects cannot be attributed to ketones alone.

(iii) Fasting-mimicking diets (FMDs) such as the Longo 5-day cycles [7,39] reproduce most endocrine and metabolic effects of fasting while preserving limited caloric intake; they induce moderate, transient ketosis [40,41]. These diets and caloric restriction (CR) in general are also associated with an extended life span in animal studies and possibly in humans [42,43].

(iv) Exogenous ketone supplementation, for example, by supplementation with BHB salts, ketone esters [44,45], or agents that increase the production of medium-chain triglycerides (MCT oils), raises circulating BHB (and sometimes acetoacetate (AcAc; a 3-oxo monocarboxylic acid anion that is the conjugate base of acetoacetic acid) levels without the concurrent suppression of glucose, insulin, or IGF-1 that characterizes the regimens (i)–(iii). About 20–30% of ingested MCT oil is converted into ketones. Studies using exogenous ketones [46], therefore, test the effects of the ketone body itself, which is decoupled from the broader endocrine-metabolic press as defined by Ref. [30]. See also Figure 1B. Conflation of these four interventions is a common source of inconsistency in the literature. Thus, researchers should carefully verify which specific regimen (or combination thereof) was used before extrapolating and comparing across studies.

### 1.3. The Lipid Metabolism Revolution in Cancer Biology

**Figure 2:**
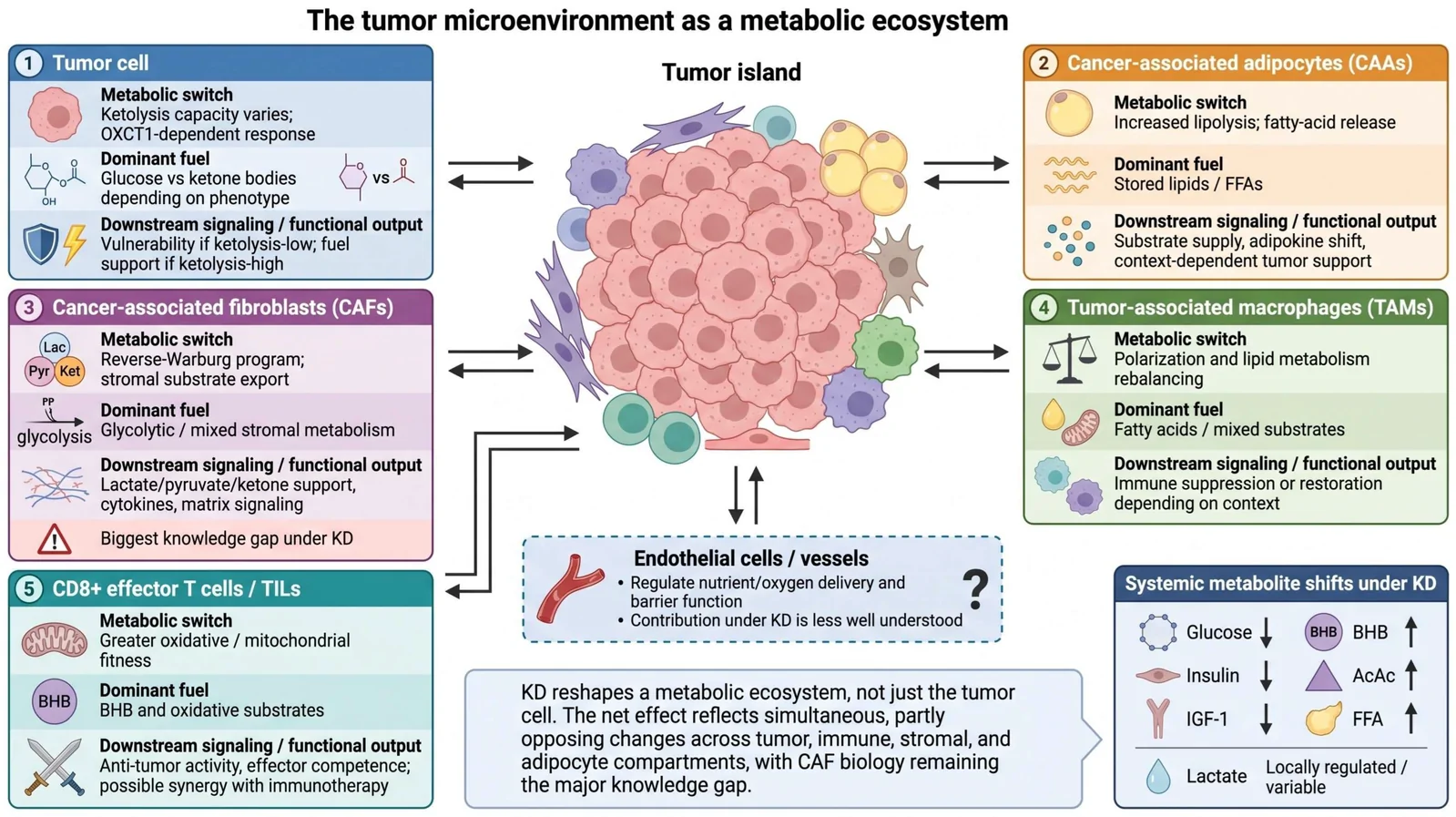
The tumor microenvironment as a metabolic ecosystem. Five main compartments and corresponding dominant cell types are recognized: (**1**) the tumor or carcinoma cells themselves, (**2**) cancer-associated adipocytes CAA, (**3**) cancer-associated fibroblasts CAFs, (**4**) tumor-associated macrophages TAMs, and (**5**) CD8+ T cells, (**2**). These cell types are shown with the metabolic switch that each may undergo when systemic ketones rise. The main message is that KD does not act on the tumor cells in isolation; multiple cell types may shift the metabolism in partly opposing directions, and the highly complex responses of multiple subtypes of CAFs remain the single biggest knowledge gap.

In parallel with clinical exploration, recent research on cancer metabolism has significantly transformed our understanding of lipid biology or lipidomics. Lipids not only provide energy through fatty acid oxidation (FAO), but they also serve as important signaling molecules. In this function, they may enable or support acquired therapy resistance and help shape the tumor immune microenvironment (TIME). A recent review [47] describes in detail how tumors and the adjacent or surrounding stromal/immune compartments may co-opt lipid uptake, e.g., via the molecule CD36, accompanied by fatty acid transport proteins (FATPs), especially the solute carrier 27A family (SLC27A1-7). Another class of critical proteins is the fatty acid-binding proteins (FABPs; [48,49,50]), including most importantly FABP4 [48] and FABP5 [51]. Lipids also push de novo lipogenesis via molecules like acetyl-CoA carboxylase (ACC1 and ACC2), fatty acid synthase (FASN), and stearoyl CoA desaturase (SCD1) [52] and the carnitine palmitoyl transferase 1 (CPT1) axis, and trigger changes in lipid droplet biology. More broadly, cancer-associated metabolic dysfunction is not limited to aerobic glycolysis: it also encompasses coordinated reprogramming of lipid uptake and synthesis, or glutamine-dependent anaplerosis (biosynthetic reactions that form intermediates of a metabolic pathway, but simultaneously use these molecules as a substrate). It further includes mitochondrial metabolism, and redox homeostasis, which all show substantial variation among tumor genotypes and different microenvironmental states [53] that may or may not be exploitable for therapeutic purposes. Consequently, glucose restriction and ketosis may expose a metabolic vulnerability in one tumor while inducing compensatory fatty acid, glutamine, or ketone utilization in another patient’s cancer. Critically, lipid metabolism operates throughout the tumor microenvironment (TME). Tumor-associated macrophages (TAMs [54]) typically accumulate lipids via CD36 or FABP4 [55], and utilize fatty acid oxidation to maintain immunosuppressive M2-like phenotypes [56,57]. Figure 2 summarizes the five most relevant cellular compartments within tumor tissues and their distinct metabolic responses to systemic ketosis.

### 1.4. The Translational Gap and Scope of This Review

A central challenge for the field is that preclinical evidence of the efficacy of ketogenic metabolic therapy has not generally translated into measurable clinical benefit. Many insights are largely drawn from in vitro experimental systems and animal (mostly mouse) tumor models [44,58], including a few genetically engineered mouse models (GEMMs). We call this issue the “lost in translation” problem, thus citing Ref. [59]. This issue is generally multifactorial. For example, it reflects both the metabolic flexibility or plasticity of human tumor cells and the highly heterogeneous, multicellular composition of the TME. Even within a single tumor tissue, cell composition and histology can vary widely (intra-tumor heterogeneity). This is naturally even more critical from patient to patient (inter-tumor heterogeneity). Accordingly, cellular heterogeneity is a common feature in all studies.

Regarding the scope of the research into lipid metabolism and KD in cancer, it is important to note that glioblastoma and other central nervous system tumors have been the subject of multiple studies [23,60], and have already been discussed in extensive reviews and meta-analyses of ketogenic metabolic therapy [61,62,63]. Thus, these papers and glioma/glioblastoma will not be a primary focus here. The present review focuses primarily on epithelial cancers, or carcinomas, including squamous cell carcinomas (SCCs) and adenocarcinomas, like pancreatic adenocarcinoma (PDAC). The carcinomas/adenocarcinomas include breast, colorectal, prostate, pancreatic, lung, ovarian, head and neck, and other epithelial malignancies. In these most frequent cancer types and subtypes, clinical evidence for the potential benefits of KD and dietary restriction is lagging behind gliomas, but is nevertheless rapidly developing. This emerging and rapidly developing field is increasingly reflected by the accumulation of evidence-based and mechanistic literature. As usual, such evidence also raises a growing number of distinct, underexplored, and even controversial translational questions, which we will try to address in this article. Glioblastoma and other CNS tumors are retained here as a metabolically instructive and relevant comparator throughout this review: a large share of the mechanistic and clinical KD evidence (Seyfried and colleagues [64], TIGER, ERGO2 studies, summarized in Table 1) originate from neuro-oncology, and the underlying biology of ketone bodies is conserved [60,65] between neuronal and epithelial cancer patients.

**Table 1:** Completed and historical clinical trials of KMT and KD in oncology. Trial-level details supporting the narrative in Section 2.3. Outcomes were reported as in the original publications; most trials lacked sufficient statistical power to detect differences in survival endpoints. n.r. = not reported in detail; SD = standard diet; ICI = immune checkpoint inhibition; HBO = hyperbaric oxygen; OS = overall survival; PFS = progression-free survival; RFS = recurrence-free survival; PR = partial response; CR = complete response. Arrows: ↑ improved status or function ↓ worsened status or reduced functionality.

Trial/Author	Tumor Type	*n*	Diet Design	Concurrent Therapy	Key Findings	Reference
Schmidt 2011	advanced (mixed)	16/5 completed 12 wk diet	KD pilot, 12 wk	none	feasibility ↑, QoL ↑, emotional functioning ↑	[66]
KETOCOMP—H&N	head and neck SCC	11/21 KD/SD	natural-foods KD vs. SD during RT; median diet duration 47 d (range 42–49) in completers; median follow-up 42 mo (range 6.7–78)	RT/RCT	OS trend (*p* ≈ 0.07), RFS trend (*p* ≈ 0.06), skin toxicity ↓ (*p* = 0.0495); BHB ↑, lean mass preserved	[67] general reviews detailing the KETOCOMP trial: [68,69,70,71]
KETOCOMP—breast	breast	32/31 KD/SD	natural-foods KD vs. SD during RT; median diet duration 35 d (range 19–47) in both arms (*p* = 0.217); data inadequate to address survival benefit	RT	numerical RFS ↑ (n.s.), BHB ↑, gradual fat loss with lean-mass preservation, stable QoL	[17,72,73]
KETOCOMP—rectal	rectal	24/25 KD/SD	KD vs. SD during chemoradiation	RCT	metabolic profile ↑, lean mass preserved; near-CR (yT0N0/yT1N0) 43% (6/14) KD vs. 15% (3/20) SD (*p* = 0.116, NS); no OS/PFS difference at 42 mo	[74]
Khodabakhshi 2020/2021	breast (locally advanced/metastatic)	80 randomized (40 KD + 40 control); 60 completed (30 + 30)	RCT eucaloric MCT-based KD vs. SD, 12 wk (single diet type, contained MCT oil)	chemotherapy	OS ↑ in MCT-KD arm (*p* = 0.04); tumor size ↓; QoL ↑; lactate and ALP ↓	[75,76]
Cohen 2018/2020	ovarian and endometrial	n.r.	RCT 12 wk KD (70:25:5)	various	visceral fat ↓, lean mass preserved, insulin sensitivity ↑, fatigue ↓	[77,78]
CAPS2	prostate	n.r.	low-carb 6 mo	active surveillance	slower PSADT correlated with ketogenesis; secondary metabolomic analysis (Chi 2022)	[79,80]
Kaiser 2024	prostate (active surveillance)	9	8 wk ad libitum KD	active surveillance	BMI ↓ 7.4%, 4/9 with no residual cancer at biopsy; no PSA change	[81]
Iyikesici 2019/2020	metastatic NSCLC and PDAC	lung *n* = 44 (2019); pancreas *n* = 25 (2020)	MSCT protocol: 12 h fast + pre-chemo insulin (Humulin R 5-20 IU) → mild hypoglycemia (2.8–3.3 mmol/L) + KD + hyperthermia + HBO	carboplatin-paclitaxel chemotherapy	tolerability acceptable; no control arm, favorable vs. historical: rectal mean OS 58.6 mo, gastric mean OS 39.5 mo vs. ~9.2 mo (V325)	[82,83]
KEATING	newly diagnosed GBM	12	modified KD vs. MCT-KD feasibility	standard-of-care RT/chemo	both feasibility arms are acceptable; proof-of-concept for DIET2TREAT	[84]
Keto-CARE (Buga 2024)	metastatic breast	20	6 mo well-formulated KD	various systemic therapies	BHB 0.7–0.8 mM target, weight ↓ ≈10%, insulin resistance ↓	[85]
ERGO	recurrent GBM	20 enrolled; 17 continued to PD; 13/17 had urine-ketone data; 3/20 (15%) discontinued early for poor QoL	unrestricted KD monotherapy	none/bevacizumab subgroup	monotherapy PFS ≈5 wk; bevacizumab + KD subgroup PFS 20 wk (6/7 objective response)	[86]
ERGO2	recurrent malignant glioma	50 randomized 1:1; 20/25 completed KD-IF (9-day CR-KD only: 3 d KD + 3 d fast + 3 d KD)	calorie-restricted KD + intermittent fasting	RT	no significant PFS/OS benefit	[60]
Schwartz 2022	GBM (case series)	12 enrolled (2 original protocol excluded; 10 revised); 9/10 (90%) revised-protocol completed 6 wk KD; 1 withdrew at 4 wk (occupational)	6 wk adjuvant KD during RT/TMZ (informal continuation in long survivors)	various long survival did not correlate with ketosis depth across the cohort, suggesting age and tumor biology, not KD duration, dominated outcomes	3/9 patients (ages 22, 28, 32 at enrollment) alive at 52, 62, and 80+ months PFS after only 6 weeks of concurrent KD	[87]
Kiryttopoulos 2024	GBM (Greek prospective)	18	Mediterranean-style KD (1:1 → 2:1–2.5:1; eucaloric; optional MCT); ≥6 mo defines adherer status	various	3 yr OS 66.7% (4/6 adherers) vs. 8.3% (1/12 non-adherers), *p* = 0.0114; adherer survival 22–82+ months	[88]
Osaka cohort (Hagihara/Egashira)	advanced solid tumors	55 (9 CRC)	modified MCT KD 1.4:1	chemotherapy	median OS 25.1 mo overall; ≥12 mo arm OS 55.1 mo vs. <12 mo 12.0 mo (4.6× gap, *p* < 0.001); 52% alive at follow-up in ≥12 mo arm	[89,90]
Furukawa (ASCO abstracts)	stage IV recurrent CRC	24 (10 + 14)	MCT KD 1.4:1	mFOLFOX6 + panitumumab/bevacizumab	ORR 60% vs. 21%; CR 50% (5/10 conversion-surgery); median OS 50 mo (responder) vs. 32.5 mo	[91,92]
Amaral 2025 phase I	newly diagnosed GBM	21 enrolled/17 evaluable (4 withdrew); 12/17 (71%) completed full 16 wk KD	16 wk 3:1 KD	standard chemoradiation	PFS 12.5 mo, OS 28.6 mo, high adherence, stable QoL	[24]
Schreck 2021	glioma	n.r.	Atkins-based KD + IF	various	84% completion; HbA1c ↓, insulin secretion ↓, cerebral ketones ↑	[93]
Vernieri 2022 (NCT03340935)	mixed solid tumors	101	cyclic 5-day FMD	standard systemic therapy	fasting glucose/insulin/IGF-1 ↓; CD8 T cell activation; IFN-γ tumor reprogramming	[41]
Ligorio 2022 (subset analysis)	metastatic TNBC and CRC	n.r.	cyclic FMD	first-line systemic therapy	exceptional/durable responses, including complete remissions in metastatic CRC	[94]
Zahra 2017	NSCLC and PDAC	9 (7 NSCLC, 2 PDAC); 3/9 (33%) completed KD	KD phase 1	RT + chemotherapy	feasibility variable, low patient compliance; tolerable in motivated patients	[95]
Tan-Shalaby 2016	advanced solid tumors (mixed)	17/11 evaluable	16 wk modified Atkins (20–40 g CHO/d)	none (no chemo)	safety/feasibility met (no SAEs); QoL preserved; only 3/11 continued past 16 wk; 2 brain-met survivors at 80 and 116 wk; weight loss ≥10% predicted response	[96] [NCT01716468]
Smith 2022 (KMT for glioma)	mixed glioma (8 GBM/gliosarcoma, 7 grade III, 1 grade II)	16	retrospective KMT + IF, mean 20.6 mo (selection: ≥4 mo KMT)	standard care (RT, TMZ, surgery, occasional TTF)	CR 8/16, PR 8/16; mean PFS 20.0 mo (subgroup GBM 13.3, grade III 17.5); cessation-rebound: 4/4 progressed within 2–4 mo of stopping; selection bias (≥4 mo inclusion)	[62]
van der Louw 2019	newly diagnosed GBM	11/6 completed	14 wk liquid KD (5–19% CHO energy)	standard chemoradiation	feasibility/safety met; 9/11 reached ketosis; KPS and coping stable; median OS 12.8 mo (range 9.8–19.0); study NOT powered to detect efficacy	[97]
Ma 2021	HNSCC	n.r.	4:1 KD phase 1	concurrent chemoradiation	low compliance (median KD 5.5 d in dropouts); paradoxical oxidative-stress markers ↑	[98]
Murphy 2024 (preclinical)	prostate (CRPC, mouse)	animal	cyclic KD + ketone augmentation	anti-PD-1 + anti-CTLA-4	response to ICI augmented; MHC class I ↑ via HDAC inhibition	[27]
Jameson 2026	metastatic PDAC	32	remotely-supervised MSKD (BHB 0.5–3 mM)	gemcitabine/nab-paclitaxel/cisplatin	OS 13.7 vs. 10.2 mo (HR 0.58, *p* = 0.107); PFS 8.5 vs. 6.2 mo (HR 0.53); PR 68.8% vs. 31.2%; no extra toxicity	[74,99]
Dantas 2026/NCT03285152	endometrial (overweight/obese, treatment-naive)	19/15 completed	VLCKD vs. SD, 21–28 d, presurgical RCT (2:1)	none (window-of-opportunity)	feasible; no grade 3/4 AEs; glucose −22%, insulin −60%; tumor p-AKT reduced; exploratory CD8+ enrichment at tumor margin; not powered for efficacy	[36]
CETOREIN study (Rolley 2026)	metastatic renal cell carcinoma	21	2:1 KD up to 12 mo, prospective pilot	systemic therapy (targeted/ICI)	feasible with acceptable tolerability in selected patients; sustained long-term adherence difficult	[100]

Literature search and scope. This narrative review was updated through 30 July 2026 using PubMed, Web of Science, Scopus, ClinicalTrials.gov, and reference-list screening. Search terms combined “ketogenic diet,” “ketogenic metabolic therapy,” “ketone bodies,” “β-hydroxybutyrate,” and cancer-related terms (“cancer,” “carcinoma,” “clinical trial,” “tumor microenvironment,” “ketolysis”). Priority was given to primary clinical and mechanistic studies in epithelial cancers; neurological malignancies were retained mainly as historically or mechanistically informative comparators. This review is narrative rather than systematic and does not follow a predefined PRISMA screening protocol.

## 2. Clinical Evidence—From Preclinical Promises to Clinical Reality

**Figure 3:**
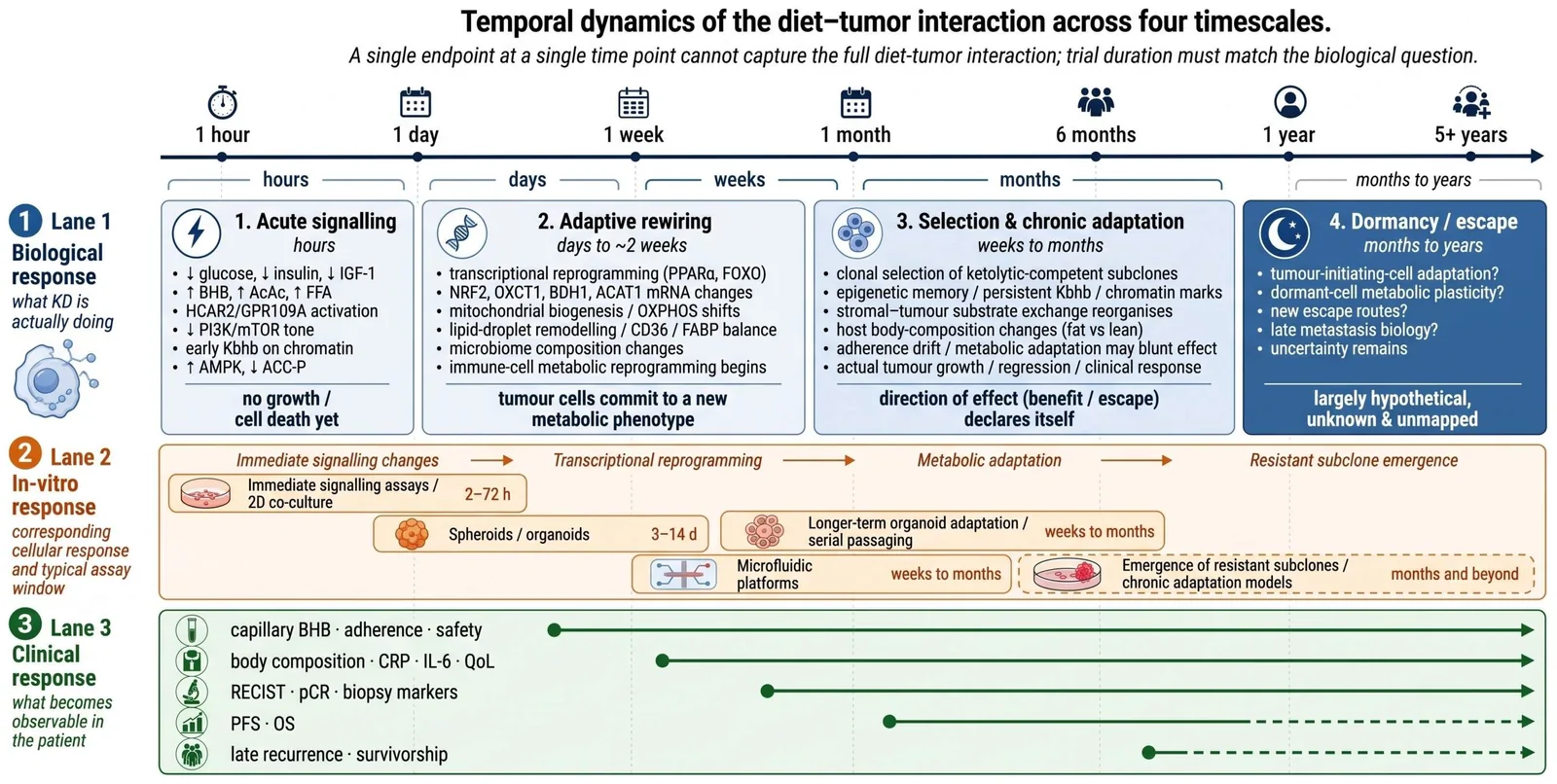
Temporal dynamics of the diet–tumor interaction across four timescales. Parallel biology, in vitro, and clinical timelines spanning hours, days, weeks, and months show that a single endpoint at any given time cannot capture the full response, that early effects may not persist, and that resistance emerges over months. As a result, both experimental in vitro studies and clinical trial duration must match the timescale of the biological question being asked.

**Figure 4:**
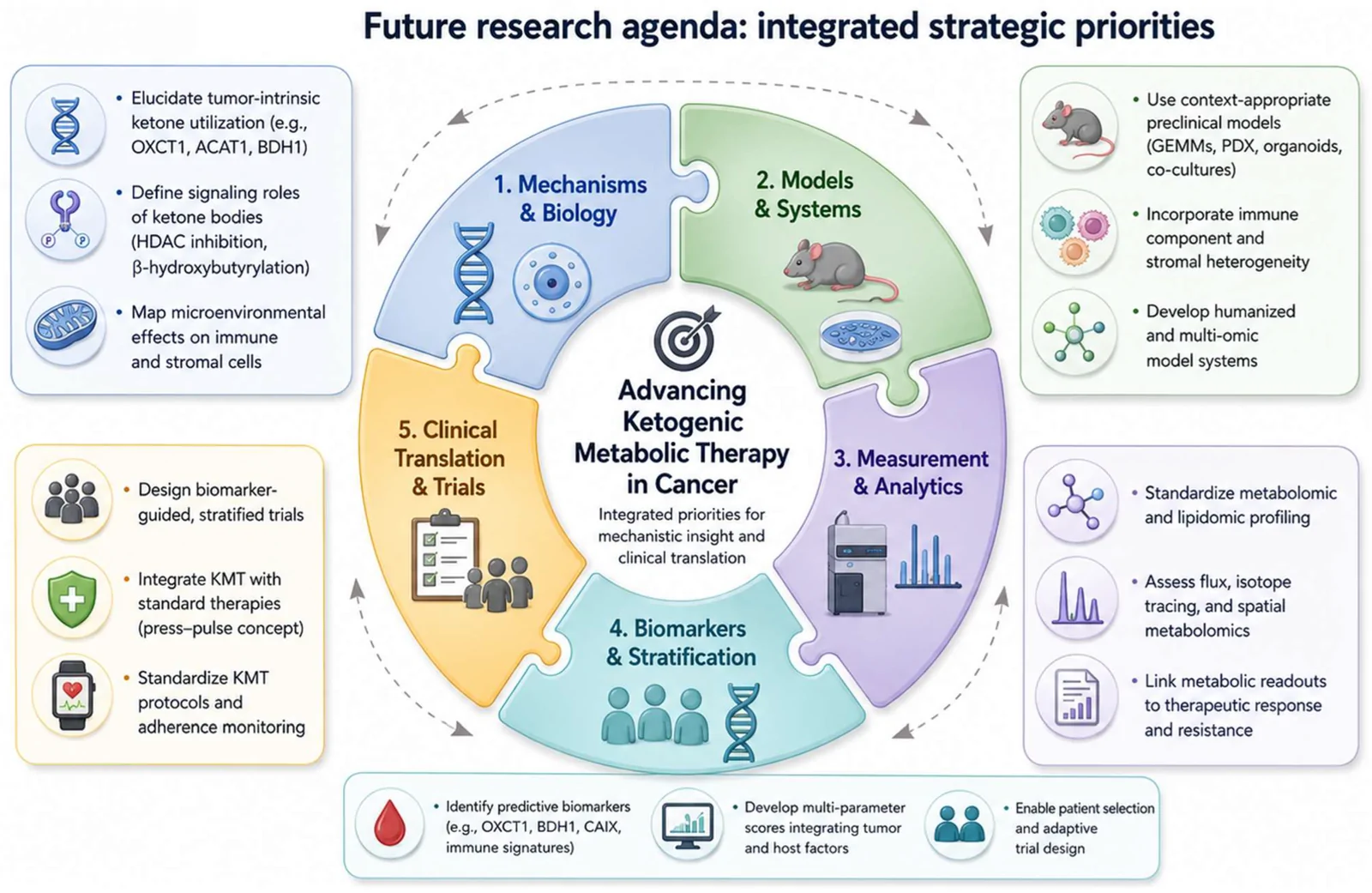
Suggestions for five testable hypotheses of the ketogenic adjuvant framework and experimental approaches for validation. Five hypotheses that follow from the review’s framework: (**1**) stratification by ketolytic capacity, (**2**) KD plus checkpoint-inhibitor immunotherapy, (**3**) BHB-versus-AcAc specificity, (**4**) the conditional role of host reserve, and (**5**) the temporal dynamics of response and resistance. In Figure 4, each of these parameters is paired with a matching experiment that would confirm or debunk or falsify them. This is a suggested, testable framework that could be beneficial for designing both future clinical trials and fundamental research experiments.

The preclinical evidence base for KD in cancer is substantial, as shown in Table 1. Across a wide range of in vitro systems and animal tumor models, ketogenic conditions have demonstrated strong anti-proliferative, pro-apoptotic, and immunomodulatory effects. As shown in Box 1, this has been achieved either through direct ketone supplementation, glucose restriction, or dietary manipulation, or combinations of these. Yet, this preclinical promise has not been successfully translated into clinical benefit and improvements in patient QoL. Understanding why this translation gap exists and stubbornly persists despite experimental work utilizing new experimental model systems, such as organoid cultures, GEMMs, and patient-derived xenografts or PDX, is probably the most consequential open question in the field [58,59,101]. Additionally, identifying the exact conditions under which clinical translation may succeed remains a major challenge. This relegates the main issue to the implementation of validated methods to improve informed patient stratification and personalized medicine (see Figure 3 and Figure 4). Figure 3 highlights the different timescales of ketogenic diet/therapy that need to be addressed in both clinical trials and in vitro experimental models.

The literature on the ketogenic diet in glioblastoma and other CNS tumors [65,86,102,103] represents a special case that has been comprehensively reviewed elsewhere [61,104]. A growing body of evidence is now accumulating for epithelial cancers, too. This includes controlled clinical trials, several small-scale early-phase 1 studies, and observational cohorts. This accumulating evidence provides a growing body of data on the feasibility, QoL benefits, metabolic response, and tumor-relevant endpoints that should be measured as the putative result of KD. For a comprehensive recent review of KD and FMD across gastrointestinal malignancies, see Ref. [105].

Another critical reorientation concerns the alleged or validated therapeutic role of ketogenic intervention, or specifically KD. The currently available body of evidence supports prioritizing ketogenic metabolic therapy for investigation as an adjuvant modality, used as an adjunct to rather than as a replacement (at least not as a monotherapy) for established standard-of-care cancer treatments. These most prominently include cytostatic chemotherapy (the “classic” anticancer drugs), radiotherapy, and immunotherapy, specifically the use of ICIs targeting PD-1 or PD-L1 molecules in the TME [106]. The therapeutic logic, grounded in the press-pulse framework introduced in Section 1.2 [30], typically rests on three pillars: (1) ketogenic metabolic priming to sensitize tumors and their microenvironments to existing standard-of-care clinical treatments [39], (2) to reduce treatment toxicity or cytotoxicity, and (3) to preserve or improve patient QoL over the treatment course [107].

### 2.1. Overview of the Clinical Trial Landscape

Clinical investigation has expanded considerably, though the field remains characterized by heterogeneous study designs, limited sample sizes, and relatively short intervention durations. A recent, comprehensive meta-analysis identified 14 controlled trials primarily from Germany, the USA, Iran, and Korea [28]. The patients studied include diverse malignancies such as glioblastoma [86], breast cancer [72,108], ovarian/endometrial cancer [77,78], pancreatic cancer [82,95], colorectal cancer (as part of the larger-scale KETOCOMP study, below), prostate cancer [27,79,80,81], head and neck cancers [67], and renal cell carcinoma [109,110]. Table 1 provides an overview of “historic” trials with a short summary of the results. This table also includes the trial on glioma and glioblastoma mentioned above. Since the total number of trials, including KMT/KD and FMD, is too large, even when focusing only on carcinomas, we must refrain here from describing each trial in much detail. As a surrogate, Table 1 provides the core data for each completed trial, with abbreviated results and outcomes.

As Table 1 illustrates, the nutritional intervention protocols vary substantially: some studies used diverse ketogenic ratios (3:1 to 4:1) [108] variable duration (4 weeks to >12 months), diverse implementation methods (dietitian-guided versus self-administered), and some combined KD/FMD with concurrent standard therapies. This heterogeneity and the lack of standards severely limit cross-study comparisons and the identification of optimal parameters for the efficacy of KD. This is also one of the reasons why the large-scale KETOCOMP study, completed several years ago in multiple phases, still provides the most reliable, rigorous methodology: its design included prespecified endpoints, a KD arm supervised by a dietician, systematic adherence tracking, and used QoL as a co-primary endpoint [109,110]. A comprehensive systematic review analyzed 39 studies (many of them also included in Table 1) involving KD/KMT, with a total of 770 patients enrolled [111]. However, the hard evidence (survival benefits, QoL) remains of relatively low quality, despite the large and growing number of trials. The typically small sample sizes, heterogeneity, and lack of common endpoints across most studies do not allow for very reliable conclusions. The Römer et al. review [111] also highlights that patient adherence rates are often poor, highly variable, and that most studies suffer from small sample sizes and methodological limitations.

Additional foundational evidence for the benefits of “freestyle” fasting and caloric restriction (CR) in oncology comes from early feasibility studies in which dedicated patients voluntarily fasted before, during, and after chemotherapy [7,8,9,39,112]. These benefits were later confirmed by a systematic review [107] concluding that fasting can reduce treatment-related toxicity, although it may come with inconsistent QoL benefits. Prolonged fasting and CR carry a serious risk of weight loss and malnutrition. Neither is generally advisable as a sustained adjunct therapy in patients who are undergoing concurrent chemo- or radiotherapy, especially when these patients are already underweight, frail, or cachexic.

### 2.2. Metabolic and Inflammatory Outcomes

The most consistent finding with KD is the achievement of ketosis, with blood β-hydroxybutyrate (BHB) levels ranging from 0.5 to 3.0 mM [113]. This can be readily achieved with adequate dietary supervision. Blood glucose reductions are similarly consistent. Inflammatory markers show particularly promising responses: C-reactive protein (CRP) showed the greatest improvement in studies lasting more than 12 weeks, a duration that now appears to be accepted as a standard in newer trials. However, the results for other metabolic parameters are less consistent. For example, insulin-like growth factor 1 (IGF-1), which is hypothesized to be reduced by ketogenic diets, shows highly variable responses, and lipid profiles vary as well. These inconsistencies may reflect significant differences in baseline metabolic status, dietary compliance, or cancer-related metabolic derangements.

Meta-analyses have now also begun to consolidate the metabolic evidence. One of these studies [114] pooled data from several controlled trials demonstrating that KD significantly reduces body weight, BMI, and fat mass, and results in significant reductions in glucose, insulin, IGF-1, and triglycerides, and increases in BHB levels. Finally, KD has been associated with improvements in emotional functioning and QoL, which may be among the most relevant issues observed across many trials. These effects were also confirmed in other studies [115], which reported overall benefits in body composition but found minimal effects on liver and kidney function, and inconsistent effects on glucose and insulin parameters. A randomized trial of patients treated for ovarian and endometrial cancer [77,78] showed that a 12-week ketogenic intervention, based on a 70:25:5% fat:protein:carbohydrate ratio, not only reduced visceral adipose tissue and helped patients preserve lean mass, but also improved insulin sensitivity (or reduced insulin resistance). In addition, the KD used in this trial significantly reduced fatigue and improved physical function.

### 2.3. Tumor Response and Survival in Carcinomas

An independent line of evidence comes from a series of randomized controlled trials (RCTs) in locally advanced and metastatic breast cancer [75]. A randomized 12-week trial that combined KD with standard chemotherapy (*n* = 80 patients) showed significantly higher global QoL and physical activity scores already at 6 weeks, and significant reductions in serum lactate and alkaline phosphatase in the KD arm by week 12. Most importantly, this arm of the study reported significantly higher overall survival (*p* = 0.04). Another publication from the same group reported significant reductions in tumor size and improvements in biochemical response at 12 weeks [76]. Several additional trials on epithelial cancers further extend this evidence base (see Table 1 for trial-by-trial details): for example, this was shown in the CAPS2 randomized trial in prostate cancer [80], the Kaiser et al. study [81], and the 12-week randomized trials on ovarian and endometrial [77,78]. Further, the colorectal arm of the KETOCOMP study [74] and trials on thoracic non-small cell lung cancer (NSCLC) [83], pancreatic adenocarcinoma (PDAC) [82], and gastric cancer [116], plus one recent study on glioblastoma (KEATING feasibility study [84]). Notably, the CAPS2 secondary metabolomic analysis revealed a bimodal PSA doubling-time distribution among prostate cancer patients in the KD arm that correlated with confirmed serum ketosis levels. This can be seen as empirical evidence that the ketosis level, rather than the assignment of a patient into one arm or another, is the central discriminator between responders and non-responders. Collectively, these trials confirm feasibility, target engagement, and safety, while leaving prospective survival benefit unproven outside the breast and pancreatic datasets discussed above.

A recent randomized feasibility study extended these observations to treatment-naive endometrial cancer [36]. Nineteen women with overweight or obesity were randomized to a 21–28-day very low carbohydrate ketogenic diet versus a standard diet, with fifteen patients completing the intervention. The diet was generally well tolerated, and produced no serious grade 3/4 adverse events. The diet substantially reduced fasting glucose levels (by approximately 22%) and insulin levels (approximately by 60%). In addition, a paired tumor analysis showed reduced p-AKT expression, which was observed together with an enrichment of CD8+ T cell signatures and increased numbers of CD8+ cells, especially at the tumor–myometrium interface. Nevertheless, intratumoral CD8 infiltration, Ki-67, and apoptosis were unchanged. As it is often the case with such studies, the relatively small cohort size and short exposure time precluded any conclusions concerning the efficacy of the diet. Nevertheless, the study demonstrates that even a brief dietary intervention can still produce measurable systemic and tumor-level changes in target and/or pathway activities in an epithelial malignancy. A separate prospective pilot in metastatic renal cell carcinoma (the CETOREIN Study) confirmed the general feasibility of such ketogenic interventions or diets during systemic therapy. The study also showed clearly that maintaining a strict 2:1 KD for up to 12 months was difficult for many patients. This suggests that shorter or more flexible regimens may be required in routine oncology, and could be ultimately more effective since compliance is encouraged [100].

One particularly informative, although preliminary analysis comes from the ongoing Osaka University Hospital program, which used a modified MCT-based ketogenic diet (1.4:1 ratio, no caloric restriction) concurrently with chemotherapy in patients with stage IV cancer. In a dedicated stage IV recurrent colon cancer subgroup of 10 patients, the KD plus chemotherapy arm achieved a 60% objective response rate, compared to only 21% in the chemotherapy-only control arm (*n* = 14), a 70% disease-control rate, and a 50% complete-response rate among the patients who proceeded to conversion surgery [91,92]. The KD-responder subgroup achieved a median overall survival of 50 months, compared with 32.5 months in the chemotherapy-only arm. Earlier initiation of the diet (~5.7 months) and higher serum ketone body levels with lower blood glucose were identified as strong predictors of response (see Table 2 for clinical predictors). The broader Osaka cohort comprised 55 advanced-cancer patients (including 9 with colorectal cancer) and reported a median overall survival of 25.1 months. Patients who maintained the KD for at least 12 months had significantly better outcomes [89,90]. These results represent some of the strongest observations yet reported for any adjuvant KD study, combined with chemotherapy in metastatic colorectal cancer. A detailed analysis of the Osaka cohort showed that patients who maintained the diet for at least 12 months had a median overall survival of 55 months, compared with 12.0 months for those who stopped earlier. This is a dramatic 4.6-fold difference (*p* < 0.001). Eight patients in the long-term group even survived for 60–121 months, demonstrating the possibility of exceptional responders.

**Table 2:** Candidate positive and negative predictors of clinical response to ketogenic metabolic therapy (KMT) in oncology. These are correlations from observational cohorts and small randomized trials, not validated biomarkers; they constitute the rationale for the shortlist of variables for prospective biomarker-stratified trials discussed in Section 3.4.1 and Section 3.4.2. References indicate the principal evidence source within this review; many predictors are supported by multiple cited studies.

Predictor	Evidence Source	Mechanism/Interpretation
**Positive predictors of clinical benefit**		
High serum β-hydroxybutyrate (BHB ≥ 0.5 mM)	Osaka cohort study; KETOCOMP study, and Keto-CARE [89] (Klement & Sweeney, 2022), [85]	Confirmed ketosis; pharmacodynamically active intervention
Low fasting blood glucose/Glucose-Ketone Index (GKI < 2)	Osaka ASCO data; Seyfried-lab framework [90]	Combined glucose–ketone balance captures Warburg suppression
KD-ABC composite (albumin ≥ 4.0 g/dL, fasting glucose ≤ 90 mg/dL, CRP ≤ 0.5 mg/dL at 3 mo)	Hagihara 2020 [89]	Validated 3-month composite for stratifying continuation
Sustained KD/KMT adherence with documented metabolic response	Egashira 2023 (Osaka long-term) [90]	Durable benefit is most often reported with continuous adherence beyond 4–6 months and frequently >12 months in case-series long-tails (4.6× OS gap in Osaka). However, radiographic and symptomatic responses can already appear within 6–12 weeks (Khodabakhshi RCT, Rieger ERGO bevacizumab subgroup). No validated minimum threshold exists; duration must be interpreted together with ketosis depth, glucose/GKI, steroid exposure, and concurrent therapy
Earlier KD introduction (~5.7 months pre-treatment)	Furukawa ASCO 2018/2019 [91,92]	Pre-treatment metabolic priming may be a critical timing variable
Elevated serum ketone bodies + TCA-cycle metabolites	CAPS2 metabolomic substudy [80]	Active ketogenesis (not just dietary low-carb) mediates the effect; bimodal PSADT response
Younger patient age (in glioblastoma)	GBM literature; Schwartz 2022 case series [87]	Older GBM patients have not derived KMT benefit in published cohorts
*Akkermansia muciniphila*/butyrate-producing gut microbiota	Glioma study [117]	Microbiome–butyrate–microglia axis; antibiotic-induced dysbiosis abolished benefit
**Negative predictors/contraindications**		
BRAF V600E mutation	preclinical melanoma [118]	Acetoacetate directly accelerates BRAF^V600E signaling and tumor growth
Advanced cachexia/sarcopenia	KETOCOMP rectal cancer arm; (Klement et al., 2021) [119]	Host metabolic dysfunction (catabolic state) must be addressed before any KD
Performance status (PS) > 2, inability to maintain oral intake, active diabetes	Osaka eligibility criteria [89]	Functional and metabolic prerequisites; minimum biological eligibility floor
Late-stage KD introduction (well after treatment initiation)	Osaka responder vs. non-responder analysis [90]	Late introduction may be insufficient to reshape tumor metabolism
High BACH1 expression in primary tumor	Preclinical breast cancer study [120]	KD may amplify metastatic potential in BACH1-high contexts; possible exclusion criterion
HMGCL/HMGCS2 high (ketolytic phenotype)	Pancreatic adenocarcinoma study [121]	Tumor uses ketones as fuel rather than starvation substrate
Concurrent broad-spectrum antibiotics	[117]	Microbiome depletion abolishes the anti-tumor effect of KD in glioma model

On the downside, direct and clear evidence for the response of tumors to KD and its impact on survival remains limited. Again, the literature on glioblastoma provides the most extensive and solid body of evidence, but remains somewhat inconclusive. A recent study summarized numerous case reports, pilots, and controlled trials, which emphasized the feasibility of KD [103], but was unable to demonstrate a definitive survival benefit for the enrolled patients. In addition, a very recent study using a 16-week 3:1 ketogenic diet with standard chemoradiation was well-tolerated by patients with newly diagnosed GBM [24]. Another recent study that is still enrolling patients with glioma [117] reported that a ketogenic intervention can inhibit glioma progression in vivo, and that this effect is likely connected to changes in the levels of butyrate produced by the gut microbiota. Critically, when the gut microbiome was depleted by antibiotics in this model, the anti-glioma effect of KD was abolished. This is a highly relevant finding with direct clinical implications, since many cancer patients receive broad-spectrum antibiotics during treatment. Patients with antibiotic-induced dysbiosis or low baseline abundance of butyrate-producing bacteria may therefore derive only limited benefit from KD, regardless of dietary adherence, motivating microbiome assessment as a candidate stratification variable.

One of the most informative single-tumor datasets on the effects of KD is probably the ERGO pilot study [86], completed in 2013 and continued in 2020 [60], in which irradiation was added. In the ERGO study, 20 patients with recurrent glioblastoma received an unrestricted ketogenic diet as monotherapy. Feasibility was acceptable, with a 15% discontinuation rate (3 of 20 patients), and ketosis was achieved in most evaluable patients [86]. However, median progression-free survival was only 5 weeks, and only a minority of these advanced patients achieved stable disease. In one arm of the study, seven patients were treated simultaneously with bevacizumab and KD. Six of these seven patients did achieve an objective response, and the median progression-free survival (PFS) with bevacizumab was 20 weeks. The study also observed a trend toward longer PFS, especially in patients who achieved more stable ketosis. This suggests that metabolic engagement via KD and fasting may be necessary, but insufficient for efficacy. This positions KD in GBM as a feasible and well-tolerated therapeutic option, but it requires combination strategies with standard-of-care clinical drugs to achieve more meaningful clinical activity.

In summary, the current clinical evidence establishes the feasibility and has shown metabolic target engagement more consistently than any measurable anti-tumor efficacy. A general overall-survival or progression-free-survival benefit, the optimal ketogenic formulation and duration for the benefit of patients, and validated predictive biomarkers all remain unproven. Future studies should therefore specify the precise interventions used (KD, fasting, fasting-mimicking diet, caloric restriction, or exogenous ketones), and should document the achieved levels of ketosis and monitor any endocrine changes. At the same time, future trials must more carefully protect patients with limited nutritional reserve, and prospectively test a spectrum of variables for more effective and precise tumor- and host-level stratification.

## 3. Determinants of Response and Translational Constraints

### 3.1. Combinations with Chemotherapy and Radiotherapy

Multiple phase I studies in radiochemotherapy contexts (see Table 1 and Table 3) consistently report that compliance is the principal limiting factor. This is readily achievable with dietitian supervision, but poor when the patient self-administers during concurrent chemoradiation. The most clinically significant results from a randomized adjuvant combination of KD with standard-of-care chemotherapy to date come from a phase II trial in metastatic pancreatic adenocarcinoma (PDAC). This study [99] randomized a cohort of 32 patients with treatment-naïve metastatic PDAC into two arms: all patients received the same standard triplet chemotherapy (gemcitabine, nab-paclitaxel, cisplatin), but were enrolled in one of two arms of the study: either with or without a remotely delivered, medically supervised ketogenic diet (MSKD). In this very recent study, nutritional ketosis was achieved in almost all patients receiving MSKD (15 of 16 in this arm). The MSKD arm also showed a longer median PFS (8.5 vs. 6.2 months; HR 0.53) and a longer median OS (13.7 vs. 10.2 months; HR 0.58; one-sided *p* ≈ 0.107), as well as a higher partial response rate (68.8% vs. 31.2%; one-sided *p* ≈ 0.096). These exploratory differences were directionally favorable, but did not establish a statistically significant survival benefit. Simultaneously, the patients showed no increase in chemotherapy-related toxicity or adverse events and no reduction in QoL [99].

**Table 3:** Ongoing and recently registered clinical trials of KD/KMT and fasting-mimicking diet (FMD) in oncology. Trial registry numbers are taken from ClinicalTrials.gov. Multiple of these trials are expected to report their first results in 2027–2028, substantially expanding the prospective evidence base. For most studies, no publications are yet available and we refer to the study entry at www.clinicaltrials.gov. - no publication available from this clinical trial.

Trial/NCT	Tumor Type	Design	Diet Arm	Combination	Status/Readout	Reference
DIET2TREAT (NCT05708352)	newly diagnosed GBM	phase II RCT, *n* = 170, 18 wk diet	KD vs. standard diet	standard chemoradiation	enrolling since 2023; first powered KD test in neuro-oncology	[24,122]
NCT06896552	advanced melanoma, cSCC, RCC	phase I/II KD + ICI, *n* = 60, 10 wk intermittent KD (2 wk on/1 wk off)	KD	immune checkpoint inhibition	enrolling at Rabin Medical Center; readout 2027–28	-
NCT06391099	metastatic melanoma, mRCC	24 wk RCT KD + ICI, microbiome co-primary endpoint (*n* = 60, 1:1 randomized)	KD (dietitian-supervised)	immune checkpoint inhibition	enrolling at OSU Comprehensive Cancer Center; readout 2027–28	-
KETOREIN (NCT05119010)	metastatic RCC	4-arm pilot, *n* = 60, 3-month KD intervention (8 wk ORR primary endpoint)	continuous KD vs. cyclic KD vs. SD ± BHB	nivolumab + ipilimumab	terminated (recruitment challenges: competing microbiome trial, refusal of dietary intervention, oral DPD intolerance); illustrates field’s high attrition rate	[123]
DIRECT2 (NCT05503108)	HR+/HER2- breast	phase III FMD vs. SOC, *n* ≈ 240, 5 FMD cycles (4 days each, every 4 wks) during neoadjuvant chemotherapy	cyclic 5-day FMD	neoadjuvant chemotherapy	trial suspended at Leiden (2024–25); illustrates the field’s high attrition rate for early-phase metabolic-intervention trials	-
BHB CAR-T (NCT06610344)	CD19+ lymphoma	phase I exogenous BHB + CAR-T feasibility (single-arm; BHB given between lymphodepletion and infusion; *n* not publicly listed)	BHB supplementation (no KD)	CD19 CAR-T cells	preclinical evidence of CAR-T metabolic enhancement; clinical trial enrolling	[124,125]
NCT06438588 (FMD-ICI)	advanced solid tumors on ICI	feasibility pilot, 4-day FMD × 3 cycles, 6 mo follow-up; primary endpoint: irAE incidence	4-day FMD cycles (Xentigen)	ongoing immune checkpoint inhibition	Recruiting at Mayo Clinic; tests whether FMD reduces immune-related adverse events	-
NCT06671613	stage IV NSCLC	FMD feasibility in front-line ICI, *n* = 66, randomized crossover, 3 FMD cycles, up to 2-yr follow-up; immune correlates	FMD	anti-PD-1/anti-PD-L1 immunotherapy	Recruiting at VA Office of Research; readout 2027	-
NCT05832086	prostate adenocarcinoma	phase 2 RCT (1:1), *n* = 138, 5-day FMD/month for 6 months; metabolic and PSA outcomes	intermittent 5-day FMD	active surveillance/standard treatment	recruiting (Freedland group; companion to CAPS2 KD work)	-
NCT07574671	early-stage breast cancer	pre-surgical window-of-opportunity; paired tumor pre/post	~3 wk KD	none (pre-surgery)	recently registered; tests KD impact on tumor microenvironment	-

### 3.2. Combinations with Immunotherapy and Fasting-Mimicking Diet

Several preclinical studies have demonstrated potential synergism between KD or fasting and immune checkpoint inhibition. In a prostate cancer model, cyclic ketogenic scheduling combined with exogenous ketone augmentation improved responses to anti-PD-1 and anti-CTLA-4 therapy [27], with mechanistic data implicating BHB-mediated HDAC inhibition and an associated remodeling of the CD8 T cell compartment. Whether this preclinical signal translates clinically is the central question of the trials summarized below and in Table 3. Three ongoing prospective trials test KD plus immune checkpoint inhibition in epithelial and skin malignancies; their key design features are summarized in Table 3. Across these ongoing trials, the central mechanistic question is whether the previously demonstrated preclinical synergism between KD and ICI [25,27,106] is mediated primarily by any of the three possibilities, or combinations thereof: (1) direct BHB-driven epigenetic remodeling of CD8 T cell effector programs, (2) by microbiome-dependent modulation of antigen presentation, or (3) by tumor-intrinsic ketolytic pathway vulnerabilities. These represent critical distinctions that the trials’ co-primary biomarker readouts (T cell phenotype, microbiome composition, tumor immunometabolism profiling) are specifically designed to resolve. Readouts from these ongoing trials are expected in 2027–2028, and will (hopefully) provide important prospective evidence on the feasibility, biological activity, and candidate biomarkers of KD-ICI combinations in “hot” versus “cold” tumor settings.

### 3.3. Counterintuitive Findings and Contexts of Caution

Beyond immunotherapy, emerging mechanistic studies start to illustrate a cautionary “context map” for future clinical translation. For example, in IL-6-producing tumor models that are typically prone to cachexia [126], persistent KD significantly delayed tumor growth, but paradoxically also accelerated the onset and severity of cachexia [127]. Wu et al. (2025) demonstrated that KD may also affect tumor stem cells, or the “stemness” of cancer-initiating cells’ [128] initiating capacity.

Finally, KD can also interact with or modulate the effects of anticancer therapies, depending on the microbiome [129]. In this study, KD reduced colonic tumor burden in a colorectal cancer model with a humanized microbiome. This was mediated, in part, by increased levels of stearate derived from bacteria in the microbiome. Likely implications for future patient stratification and a list of candidate biomarkers are summarized in Section 3.4 and in Figure 4.

### 3.4. Critical Limitations and Host-State Considerations

**Figure 5:**
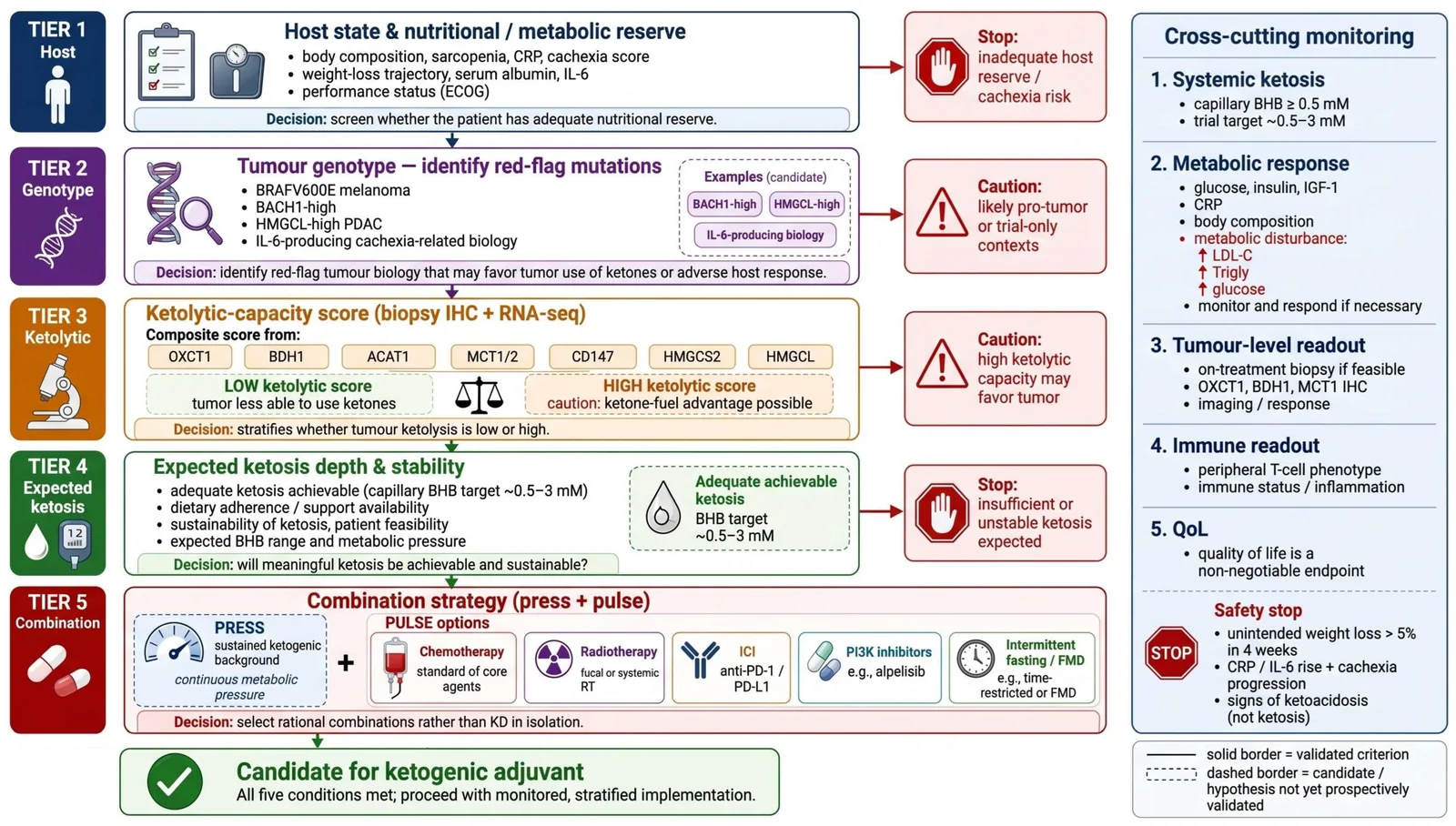
Tiered, hypothesis-generating framework for candidate patient stratification in ketogenic intervention trials. Five sequential levels (tiers) can be explored for informed decision-making in metabolic trials: (**1**) host reserve, (**2**) tumor genotype, (**3**) ketolytic capacity, (**4**) evidence of achievable ketosis, and (**5**) possibilities for combination strategy. These tiers/levels identify candidate variables for prospective testing of patient stratification; they are hypothesis-generating and should not yet be interpreted as solid, validated clinical eligibility criteria. The solid versus dashed borders in the figure relate to the most relevant candidate-specific metabolic criteria and indicate if they are firmly established or not yet established (candidates). QoL is generally retained as a non-negotiable and desired endpoint.

Clinical studies cannot discern direct effects contributed by different mechanisms: the most likely mechanisms include (1) altered substrate availability in tumors with limited metabolic flexibility, (2) endocrine effects mediated by reduced systemic insulin/IGF secretion and signaling, (3) immune and inflammatory remodeling, (4) microbiome-mediated effects, or (5) the positive contributions of weight loss and caloric restriction. Along these lines, multiple mechanistic studies were able to demonstrate that ketogenic conditions do not necessarily “starve” the tumor, but can even promote tumor growth, at least in certain, specific contexts. For example, one study showed that in melanomas positive for the BRAF^V600E mutation, a diet-driven increase in acetoacetate (AcAc) levels promoted tumor growth rather than reducing it. This occurred in a mutation-dependent manner [118]. In an in vivo metastasis model, sustained KD unexpectedly enhanced BACH1-mediated metastatic programs (BTB and CNC Homology 1 transcription factor) [120]. Additionally unexpected, it was shown that β-hydroxybutyrate or BHB can sometimes promote metastasis and not block it. This occurs, for example, via β-hydroxybutyrylation-dependent stabilization of the transcription factor Snail [130]. PDAC can also exploit ketone metabolism for its benefit, rather than suffer from it. It was shown that the enzyme HMG-CoA lyase (HMGCL), in combination with increased levels of β-hydroxybutyrate, effectively supported tumor progression in PDAC [121], contrary to what one might have expected. The context map shown in Figure 5 visualizes the molecular and metabolic background for these discrepancies and inconsistencies. It also suggests several control and decision points for informed decision-making, based on the “benefit versus uncertainty or harm” criteria, which can be used in clinical settings to group or stratify patients according to the expected direction of effect. Such monitoring issues may not have been strictly observed in many trials, but should be carefully considered.

A comprehensive mechanistic review [25] concluded that KD may sensitize at least some cancers to anticancer therapy and has an overall favorable safety profile; however, we still cannot be certain that this will translate into definitive clinical efficacy. This uncertainty requires controlled trials with strongly standardized protocols. Another study further elaborates on this unmet need [131]. This study suggests that a more critical assessment of patients prior to enrolling them in trials is needed (see Figure 5). They emphasize that, despite promising preclinical data, clinical trial results may remain uncertain and often yield surprises (hence the term “trials”). Especially in KD and related therapies, there are still substantial gaps in our understanding of which patients or patient populations may benefit from KD, which fasting and/or KD protocol is optimal, and which intervention parameters are likely to be optimal. In future trials, this will also require more careful measures applied across patient cohorts, such as stratification by ketolytic capacity, KD plus checkpoint-inhibitor immunotherapy, BHB-versus-AcAc specificity, the conditional role of host reserve, and the temporal dynamics of response and resistance (Figure 4). This figure includes experimental measures to confirm metabolic measurements in both clinical and experimental studies.

The nutritional risk constraint is underscored by direct evidence that nutritional adequacy itself affects survival. One recent study [119] showed that individualized nutrition therapy that specifically targets energy balance significantly improved the 12-month overall survival in cachectic cancer patients (73% survived, compared to 47% in controls), providing strong experimental evidence that the metabolic state of the host (the cancer patient) itself is a therapeutic target, not just the tumor. This creates a fundamental tension: strategies for energy restriction may harm some vulnerable patients who are already catabolic, even if the same strategies may benefit metabolically healthier, younger, less advanced patients. The Osaka program [89,90] utilized this principle through explicit eligibility criteria: PS or performance status ≤ 2, retained capacity for oral intake, and absence of active diabetes.

#### 3.4.1. Response Heterogeneity and Long-Tail Outcomes

The estimates provided in this subsection should be interpreted with caution. Because they are derived largely from retrospective and observational data, any longer dietary exposure may be a consequence of longer survival rather than its cause. In addition, the depth, duration, and adherence of patients to ketosis-inducing diets are best regarded as candidate exposure variables rather than validated predictive biomarkers.

Outcome variability in KD trials appears to have two separable components: (i) general heterogeneity is at the level of disease biology. Then, there is also a second component (ii), specific to long-term ketosis trials, that becomes visible only when adequate ketosis is achieved and maintained for a sufficient duration. Two contrasting trials illustrate this. In the original ERGO study [86], KD monotherapy for recurrent glioblastoma showed a tightly clustered distribution with no clear long-term responders. In contrast, a small subgroup of patients who received KD plus bevacizumab (an anti-VEGF antibody) achieved six of the seven objective responses. These patients also achieved a maximum PFS of over 120 weeks. This is well beyond the upper end of historical cohorts treated with bevacizumab alone. This exploratory, non-randomized subgroup raises the possibility that KD may modify sensitivity to selected anticancer therapies, but it is too small to firmly establish a significant treatment interaction. By contrast, the KETOCOMP rectal cancer cohort [74] showed an extremely broad follow-up range of 1.5–127.1 months. This extreme distribution is not differentiated between KD and standard-diet arms, and no survival difference was detected. Again, the broad range may reflect intrinsic disease heterogeneity rather than a KD-specific impact. The same logic applies to the stratification observed in the Osaka group trials, driven prominently by KD duration [89,90]. Here, the patient group that strictly adhered to KD for over 12 months showed both a markedly higher median overall survival and a distribution that showed a characteristic long upper tail. This was not observed when compared to the short-term KD group (see also paragraph Section 2.3 and Table 1). A possible conclusion is that separating intrinsic heterogeneity that is attributable to biology and tissue (or cellular) composition from the enrichment of KD-specific super-responders requires additional stratification, for example, based on confirmed ketosis exposure (see Figure 4 for corresponding measures and testing). The mere assignment to a particular arm of the study is not sufficient. Quantitatively, in the Osaka long-term cohort, approximately 20–25% of unselected advanced cancer patients appear to represent possible long-term beneficiaries or even super survivors. Their overall survival exceeded 60 months, far exceeding the 2–3-month median benchmark for newly approved cytotoxic agents discussed in Section 1.1.

A complementary distributional reading concerns the duration dimension itself. Across the primary trials with patient-level outcome data, the prescribed KD spans an enormous range—from 9 days (ERGO2 KD-IF schedule [60] to 12–16 weeks of formal protocols [24,75], and indefinite continuation in retrospective cohorts [62,88]. The shortest documented effective duration in a randomized trial is 12 weeks [75,76], applied to patients with locally advanced/metastatic breast cancer. There have been intense discussions centered on these findings [132,133] continued in a recent review article [134] by the same authors. Generally, longer KD adherence correlates with longer survival in observational GBM and the Osaka data, but these correlations cannot be cleanly separated from survivor-time bias: patients who live longer naturally accumulate more KD-months, almost by definition. Consequently, the optimal KD duration for patients enrolled in trials remains unanswered. Current data do not support a strict ≥ 12-month threshold as a prerequisite for benefit; rather, they suggest a minimum exposure of ~12 weeks during the active treatment window. Thus, a minimum duration of 12 weeks, with continued exposure thereafter where it is feasible, is the most defensible empirical recommendation.

A particularly informative observation comes from a retrospective report about a glioma series [62]: among the patients with documented favorable responses on KMT, all four who voluntarily discontinued the diet experienced radiographic progression within 2–4 months of cessation. Two patients regained control after re-instituting KMT. Although certainly limited by a small sample size and selection bias, the cessation-rebound pattern observed in this trial supports the temporal dynamics argument already discussed above (Figure 3). It also implies that ketogenic intervention should perhaps be conceptualized as a sustained or cyclic exposure rather than a discrete induction phase.

#### 3.4.2. Tumor Metabolic Phenotype as a Pre-Treatment Stratifier

**Figure 6:**
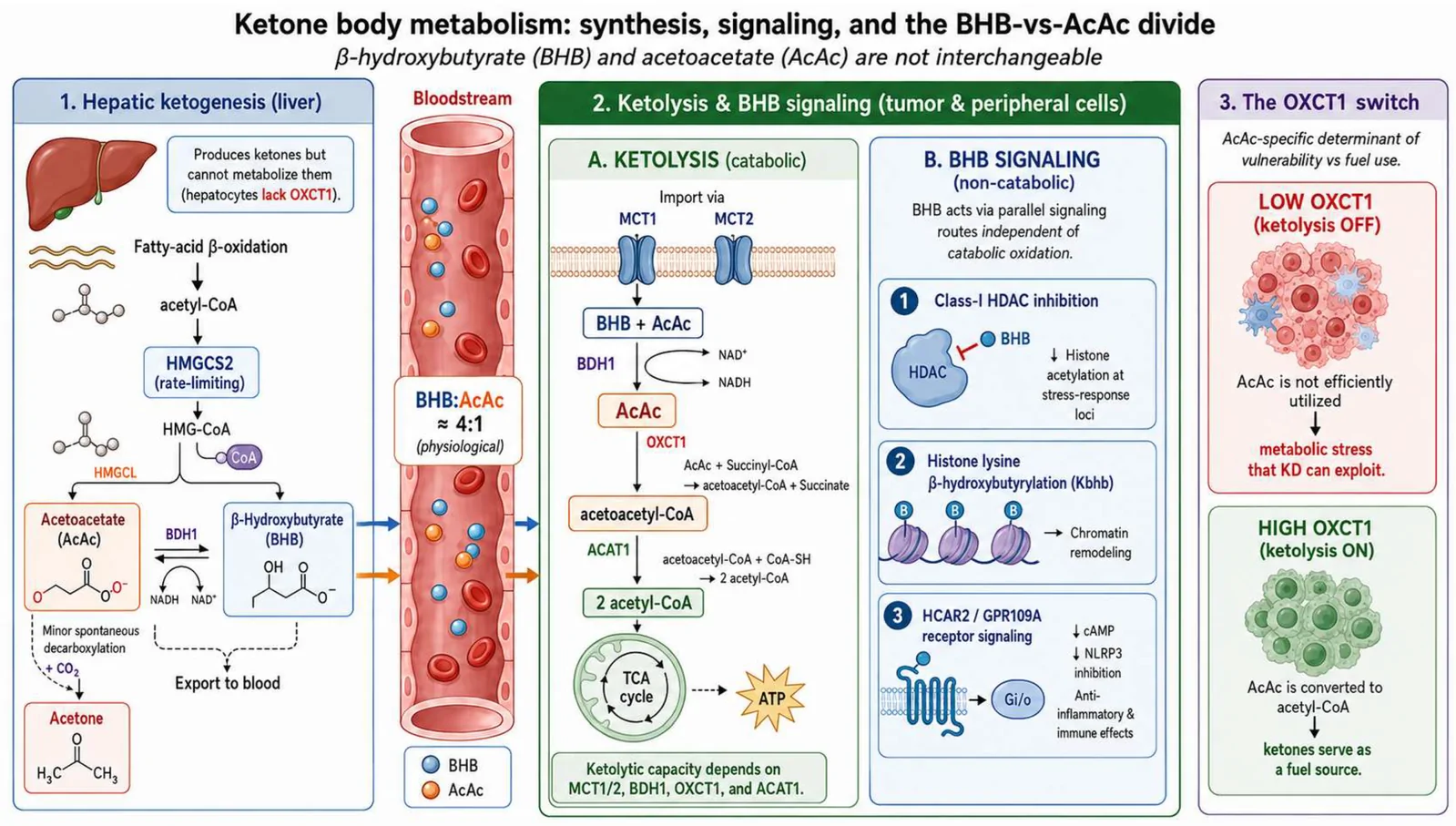
Ketone body metabolism: synthesis, signaling, and the BHB-vs-AcAc divide. (**1**) Hepatic ketogenesis exports BHB and AcAc (≈4:1) that (**2**) peripheral and tumor cells take up via MCT1/2 and metabolize through BDH1, OXCT1, and ACAT1. BHB also signals independently through class-I HDAC inhibition, histone β-hydroxybutyrylation, and HCAR2/GPR109A. The OXCT1 switch (**3**) determines whether ketone bodies represent a metabolic stress factor (low OXCT1) or are used as a fuel (high OXCT1). This renders the ketolytic capacity of cancer cells and tissues a measurable, stratifiable property.

A complementary stratification axis to the host-state and ketosis-exposure variables discussed above is the tumor’s intrinsic metabolic profile. Three broad metabolic phenotypes of tumors can be commonly distinguished using readily available preclinical and clinical markers. These three groups may differ in their predicted response to ketogenic intervention: (1) Glucose-dependent, high-Warburg tumors, characterized by elevated activity visible in ^18^F desoxyglucose (^18^F-FDG) positron emission tomography (PET), high lactate dehydrogenase (LDHA) expression, low MCT1 expression, and reduced oxidative-phosphorylation (OXPHOS) capacity. These tumors represent the canonical KD candidates. The limited substrate flexibility and “addiction” to glucose make them theoretically vulnerable to glucose restriction; the ideal situation. (2) The metabolically flexible tumors. These tumors are, in principle, capable of switching among glucose, glutamine, and fatty acids as substrates. Such tumors may not respond to KD alone, or not at all. However, pairing KD with metabolic co-targeting (e.g., glutamine antagonism via 6-diazo-5-oxo-L-norleucine (DON) and its prodrugs may represent an option. The use of DON in combination with KD is a rational extension of the therapy for these types of tumors. (3) Finally, there is a subgroup of ketone-using tumors, expressing high levels and activities of mitochondrial 3-hydroxy-3-methylglutaryl-CoA synthase 2 (HMGCS2) and 3-oxoacid CoA-transferase 1 (OXCT1). These same tumors also tend to have intact MCT1 and OXPHOS activities. Figure 6 (right panel and OXCT1-switch inset) makes this dichotomy explicit at the enzyme level. Such tumors can use ketones as fuel rather than starving on them. Several cancer subtypes have been identified, characterized by high expression of BACH1 [120] or 3-hydroxy-3-methylglutaryl-CoA lyase (HMG-CoA lyase, HMGCL) [121]. Another group of ketone-avid tumors is characterized by the prevalence of BRAF^V600E mutations in melanoma [118]. The existence of such “ketone-utilizing tumors” requires their explicit identification and subsequent elimination from any trials that employ KD protocols. Testing for these phenotypes prior to patient enrollment in ongoing trials would require a complex pre-treatment examination protocol that ideally combines FDG-PET/CT with tumor biopsy and immunohistochemistry (IHC) staining for relevant markers such as HMGCL, BACH1, and HMGCS2. As of this writing (June 2026), no published KD/KMT trial has prospectively stratified its patient cohorts by this type of tumor metabolic profiling.

## 4. Molecular Mechanisms

### 4.1. The Warburg Effect: Metabolic Reprogramming or Mitochondrial Dysfunction?

The Warburg effect describes the observation that cancer cells convert large amounts of glucose to lactate even in the presence of oxygen [14]. Even though aerobic glycolysis is less efficient at producing ATP, it gives cancer cells several advantages, such as much faster glucose uptake and processing, which supports rapid cell division. This speed advantage can outpace respiration when proteome capacity is limiting. As a result, aerobic glycolysis, rather than oxidative phosphorylation (OXPHOS) of glucose, produces or retains more building blocks for biomass, such as nucleotides, amino acids, and lipids, which in turn fuel tumor expansion. Furthermore, the Warburg effect acidifies the TME via lactate secretion, thereby promoting tumor cell invasion and immune evasion. It further supports angiogenesis and resistance to therapy. In short, the Warburg effect helps tumors survive under various hostile conditions. The Warburg effect is now considered a key metabolic hallmark of cancer, shaping how tumors grow, spread, metastasize, and how they may resist treatment. It is a metabolic shortcut that trades energetic efficiency for speed and much greater flexibility, providing multiple survival advantages at once. All of this makes the Warburg effect a central driver of tumor progression [135] and is metabolically linked to KD and fasting.

Warburg originally proposed that this phenomenon results from impaired mitochondrial respiration and that defective oxidative phosphorylation is the primary cause of cancer. In his view, enhanced glycolysis is compensatory, arising because damaged mitochondria cannot meet cellular energy demands. Thomas Seyfried and coworkers have extended this argument. They propose that cancer is fundamentally a mitochondrial metabolic disease in which genomic instability and oncogenic mutations arise downstream of chronic respiratory dysfunction and redox imbalance [30,64].

An alternative interpretation holds that the Warburg effect does not primarily reflect defective mitochondria, but rather the metabolic requirements of rapid proliferation. In this view, most cancer cells retain functional mitochondria and active oxidative phosphorylation. Aerobic glycolysis is also observed in normal, noncancerous, highly proliferating cells, such as activated lymphocytes and certain embryonic tissues. The metabolism of proliferating cells is adapted to facilitate the uptake and incorporation of nutrients into biomass: nucleotides, amino acids, and lipids are all needed to generate a new cell. Glycolytic intermediates are therefore diverted into anabolic pathways rather than being fully oxidized to yield maximal ATP. Importantly, ATP may never be limited in these cells; proliferating cells often maintain high ATP/ADP ratios despite reliance on aerobic glycolysis [13]. Thus, the apparent “inefficiency” of glycolysis is not a liability, but a strategy that prioritizes biosynthesis and growth over energetic efficiency. Oncogenic signaling pathways such as PI3K/Akt, mTOR, MYC, and HIF-1α reinforce this metabolic program by coordinating nutrient uptake and enzyme regulation to support biomass production and cell division.

Current evidence suggests that these models are not mutually exclusive. Many tumors exhibit intact but rewired mitochondria that support both oxidative metabolism and biosynthesis, while metabolic stress and altered redox states can contribute to genomic instability. Cancer metabolism appears highly plastic, shaped by interactions between oncogenic signaling and mitochondrial function. This opinion was reviewed almost 20 years ago by Refs. [136,137]. For ketogenic therapy, which is also an oncometabolic therapy, this integrated view implies that restricting glucose uptake, glucose levels, and insulin signaling may impose metabolic stress on tumor cells that are constrained by anabolic and redox demands. This is regardless of whether mitochondrial dysfunction is primary or secondary in tumor development [135].

### 4.2. Ketone Body Metabolism: Beyond Energy Substrates

Ketone bodies β-hydroxybutyrate (BHB), acetoacetate (AcAc), and acetone itself are synthesized in liver mitochondria during carbohydrate restriction or fasting. The pathway begins with acetyl-CoA from fatty acid β-oxidation. The enzyme 3-Hydroxy-3-Methylglutaryl-CoA Synthase 2 (HMGCS2) catalyzes the rate-limiting step, producing 3-Hydroxy-3-methylglutaryl-CoA (HMG-CoA), which is cleaved to acetoacetate. Most of the acetoacetate is then reduced to BHB by 3-Hydroxybutyrate Dehydrogenase 1 (BDH1), but a fraction spontaneously decarboxylates to acetone. Ketolysis occurs in extrahepatic tissues where BHB is oxidized back to acetoacetate by BDH1, then activated by 3-Oxoacid CoA-Transferase 1 (OXCT1). This represents an important, rate-limiting step that occurs via transfer of CoA from succinyl-CoA. The resulting acetoacetyl-CoA is finally cleaved by Acetyl-CoA Acetyltransferase 1 (ACAT1) to yield acetyl-CoA for the TCA cycle. Critically, OXCT1 is not expressed in hepatocytes, which explains why the liver produces ketone bodies but cannot consume them.

Beyond serving as a metabolic fuel, BHB functions as a potent signaling molecule [138]. In 2013, it was demonstrated that BHB is an endogenous inhibitor of class I HDACs [139], thereby increasing histone acetylation at loci associated with oxidative stress responses. At physiologically achievable concentrations (~1–5 mM), BHB couples systemic ketosis to changes in the chromatin state and epigenetic gene regulation. Most importantly, in 2016, BHB has also been identified as a substrate for lysine β-hydroxybutyrylation (Kbhb) of histones, which is distinct from acetylation induced by HDAC inhibition [19]. For example, lysine β-hydroxybutyrylation promotes cancer metastasis through Kbhb-dependent stabilization of the transcription regulator Snail [130]. Several recent reviews provide up-to-date overviews of the roles of lysine β-hydroxylation, particularly in cancer [18,20], and describe how the ketogenic diet reshapes cancer metabolism through Kbhb modifications [20].

BHB further functions as a ligand for Hydroxycarboxylic Acid Receptor 2 (GPR109A, or HCAR2). GPR109A activation by BHB induces anti-inflammatory effects within the tumor stroma and tumor tissue. This is mediated through inhibition of the NLRP3 inflammasome (NLR Family Pyrin Domain Containing protein 3), and it was shown that BHB effectively blocks NLRP3-mediated inflammatory disease [21]. In addition, it was demonstrated that the anti-tumor effects of a ketogenic diet were partly abolished by GPR109A antagonism [106]. A complementary pathway specific to epithelial cancers was described later [113], in which the ketogenic diet first induced BHB signaling via GPR109A/HCAR2. This, in turn, activated the transcriptional regulator HOP Homeobox protein (HOPX), effectively suppressing intestinal tumor growth in mouse models of colorectal cancer.

**Figure 7:**
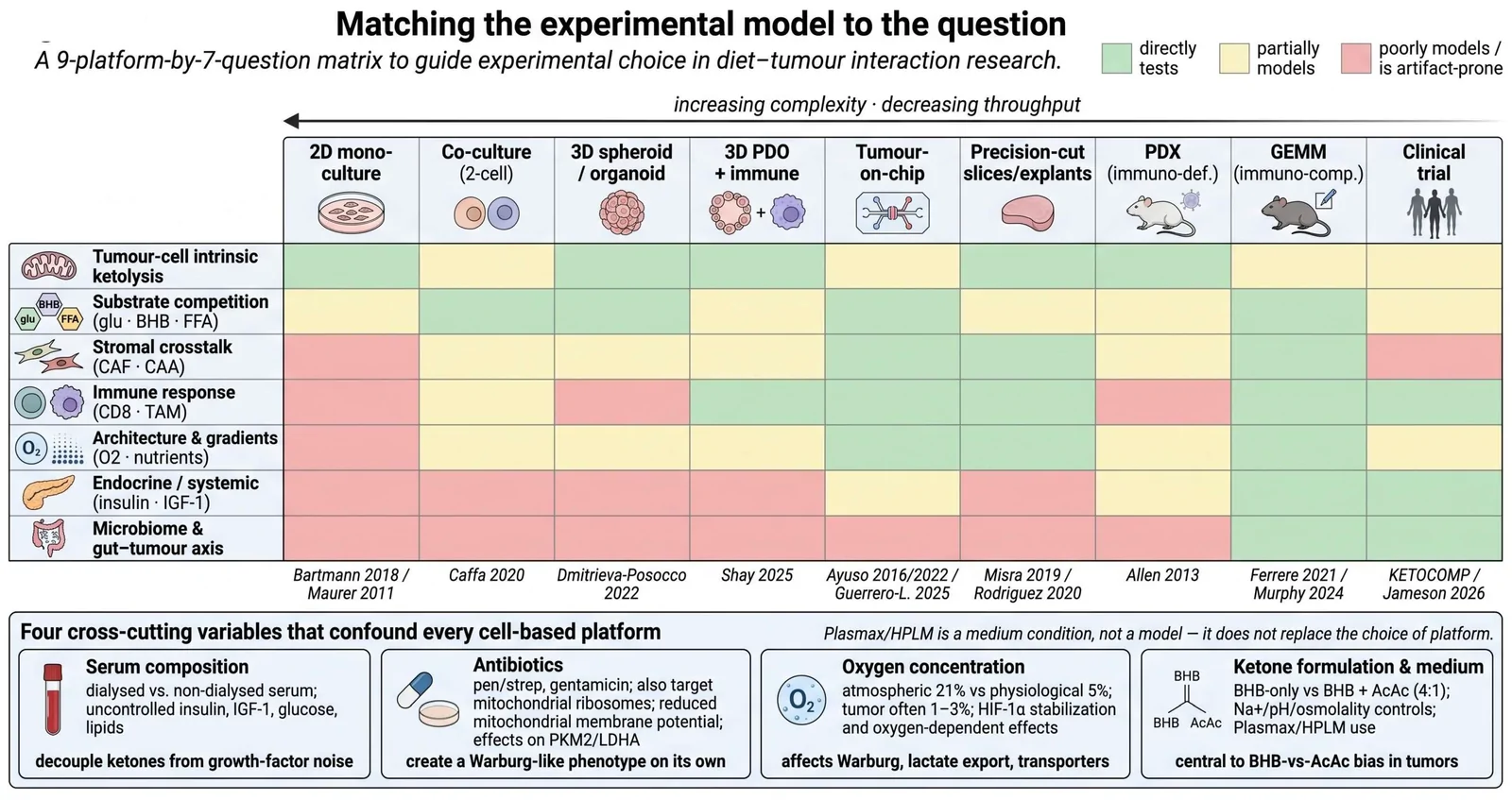
Matching the experimental model to the question. A platform-by-question matrix mapping models (2D mono- and co-culture, 3D spheroids/organoids, PDOs, tumor-on-chip, tissue slices, PDX, GEMM, and clinical trials) against the mechanistic questions each can address, with serum composition, antibiotics, oxygen tension, and ketone formulation flagged as cross-cutting confounders. No single platform answers all questions; triangulation across platforms is required.

While BHB has dominated the recent literature, its interconvertible counterpart, acetoacetate (AcAc), exerts distinct effects that are frequently overlooked. AcAc can directly inhibit cancer cell proliferation through a UCP2-dependent reduction in ATP production and synergistically enhance chemotherapeutic cytotoxicity. Unlike BHB, AcAc is the obligate substrate of ketolysis via OXCT1 in extrahepatic tissues, which makes OXCT1 expression the pivotal switch determining whether AcAc acts as a metabolic vulnerability: in OXCT1-low tumors, AcAc accumulates, and its anti-proliferative effects dominate. In contrast, AcAc can also be used as an efficient fuel. This, specifically, is observed in OXCT1-high tumors: AcAc is consumed by these cancer cells to regenerate acetyl-CoA, and it may even support aggressive behavior, as documented in prostate cancer. Two practical implications follow from this switching: First, any in vitro ketogenic medium should contain both BHB and AcAc (roughly 4:1 in physiological ketosis) rather than BHB alone. Second, OXCT1 expression, together with BDH1, ACAT1, and HMGCS2, should be profiled upfront as a patient stratification biomarker (see also Figure 6 and Figure 7) and also in experimental settings.

### 4.3. Cancer Cell Metabolic Flexibility and Vulnerability

The potential for ketogenic diets to selectively disadvantage cancer cells largely relies on the correct interpretation of the Warburg hypothesis: cancer cells become increasingly dependent on aerobic glycolysis, they are less able to adapt to glucose restriction, and they begin to utilize ketones. In fact, the key enzyme 3-Oxoacid CoA-Transferase 1 (OXCT1) is not only the rate-limiting enzyme of ketolysis, it has also emerged as a key determinant of tumor response to caloric and glucose restriction. High OXCT1 expression is typically found in the tumor cells themselves, not the stroma, and it is typically associated with aggressive behavior, at least in prostate cancer [140,141]. In contrast, low OXCT1 expression in glioblastoma has been associated with a better response to ketogenic intervention [63,142]. This molecular stratification suggests that ketolytic enzyme profiling could guide patient selection [103].

Recent work indicates that OXCT1 is not only a mitochondrial determinant of ketolytic capacity, but may also govern locus-specific epigenetic and immune responses. In hepatocellular carcinoma (HCC), glucose deprivation induced AMPK-dependent phosphorylation of OXCT1 at S113 and its translocation to the nucleus. Once in the nucleus, p-OXCT1 associated with IRF1, resulted in locally depleted levels of BHB, and reduced H3K9 beta-hydroxybutyrylation at MHC-I and diverse chemokine loci. As a result, this led to suppressed antigen-presentation and immune-recruitment genes. High tumor OXCT1 levels also correlated with poor immune-checkpoint-blockade response. In contrast, interfering with the AMPK-OXCT1-IRF1 axis improved sensitivity to checkpoint inhibitors under ketogenic conditions [143]. Additionally in HCC, a defined oncogenic genotype based on tumor cells with mutant beta-catenin, transcriptionally activated OXCT1 expression and was able to effectively reprogram ketone body metabolism. As a result, this was then driving increased metastasis and resistance to ketogenic therapy [144]. These very recent findings promote OXCT1 beyond a simple paradigm that reads like “low OXCT1 = KD-sensitive tumors, high OXCT1 = ketone-fueled tumors.” The new data suggest a slightly more advanced model in which high levels of OXCT1 expression and metabolic activity are shaped by genotype, subcellular localization, and immune context. These observations are currently derived exclusively from HCC, and they should not yet be generalized to all of the epithelial tumors or carcinomas.

Cancer cells also exhibit profound alterations in lipid metabolism that extend far beyond the glucose-centric focus of the Warburg effect. These encompass fatty acid uptake, e.g., via CD36 (also known as platelet glycoprotein 4 or fatty acid translocase), long-chain fatty acid transport proteins (FATPs), including the SLC27A family of solute carriers, and fatty acid-binding proteins such as FABP4 and FABP5 [48,55,145]. Other key enzymes differentially expressed in tumor cells are ATP Citrate Lyase ACLY [146] and Acetyl-CoA carboxylase ACC. Furthermore, tumor cells differentially engage in fatty acid modification, including expression of stearoyl-CoA 9-desaturase (SCD1 [52]) and fatty acid elongases (ELOVL family). Under ketogenic conditions, when glucose-derived citrate for lipogenesis is limited, cancer cells may become more dependent on exogenous fatty acid uptake. For example, CD36 expression has been shown to mark metastasis-initiating cells and correlate with poor outcomes [147]. Importantly, SCD1, FABP4, and CD36 expressed on CD8+ T cells promote lipid uptake, potentially leading to ferroptosis [148], and thus impairing anti-tumor immunity. However, the same enzymes, when overexpressed in tumor cells, have also been shown to confer resistance to ferroptosis, thus promoting recurrence [52]. The situation, therefore, is not straightforward, but highly complex and probably driven by tumor heterogeneity and plasticity. We can only conclude that the lipid-rich environment in the tumor stroma, potentially induced by ketogenic diets, may exert complex, cell-type-specific effects, and that these may be either beneficial or detrimental, depending on the context. It is also clear that further research is needed to translate these findings into improved patient stratification and personalized medicine. We should also cover other metabolic enzymes in this context.

MCT1 (monocarboxylate transporter 1, SLC16A1) does not merely shuttle lactate and pyruvate across the plasma membrane. MCT1 is also a critical component of a supramolecular complex embedded in the inner mitochondrial membrane, the mitochondrial Lactate Oxidation Complex (mLOC). This complex enables direct mitochondrial oxidation of lactate, fundamentally revising the classical view that only pyruvate (and not lactate) serves as the glycolytic substrate entering the TCA cycle. The consequences affect multiple aspects of metabolism: partitioning of energy substrates, redox homeostasis, mitochondrial biogenesis, and the metabolic symbiosis between oxidative and glycolytic tumor subpopulations. For KD/ketone biology, this is directly relevant because ketone oxidation converges on the same mitochondrial acetyl-CoA pool. MCT1 is shared as the plasma membrane importer for both lactate and BHB. Consequently, MCT1/CD147 blockade (e.g., by crizotinib) simultaneously disrupts lactate shuttling and ketone import. This may explain the sensitization of tumor-initiating cells by ketone bodies, as reported by Ref. [128].

### 4.4. Connecting Ketogenic Conditions to Lipid Reprogramming

The shift to ketogenic metabolism creates a fundamentally altered metabolic environment intersecting with cancer lipid reprogramming at multiple levels: (1) Reduced glucose availability limits de novo lipogenesis by depleting the citrate pool. (2) Elevated ketone bodies in the blood provide an alternative source of acetyl-CoA for (tumor) cells that are capable of ketolysis and simultaneously function as signaling molecules. (3) Lipid reprogramming can result in reduced secretion and lowered systemic levels of hormones and growth factors like insulin and IGF-1, which is balanced by elevated glucagon secretion. This shifts the balance from lipogenic to more lipolytic and lipid oxidative programs. (4) Lipid preprogramming also provides an environment with increased systemic fatty acid availability, mainly from enhanced adipocyte lipolysis.

These changes likely have many cell-type-specific consequences. Cancer cells with high glycolytic flux and limited ketolytic capacity may experience a metabolic crisis and are vulnerable to stress. In contrast, cancer cells with metabolic flexibility may be adapted by increasing FAO and ketone utilization. Adipocytes and cancer-associated adipocytes (CAAs) may increase lipolysis, thereby providing fatty acids to neighboring cells. Cancer-associated fibroblasts (CAFs) may shift metabolic support from lactate to other substrates. Finally, infiltrating immune cells, such as tumor-associated macrophages (TAMs) or tumor-infiltrating lymphocytes (TILs), may be reprogrammed toward anti-tumor or immunosuppressive phenotypes depending on their metabolic dependencies.

The differential stress resistance/sensitization (DSR/DSS) framework [149], introduced by Valter Longos’ group in 2008, provides perhaps the strongest mechanistic bridge between metabolic intervention and therapy response. A 48 h short-term starvation in colon cancer models produces an “anti-Warburg” metabolic shift, resulting in decreased glycolysis, increased uncoupled respiration, and oxidative stress. This leads to apoptosis, which synergizes with oxaliplatin [150]. It was shown that fasting cycles retard tumor growth and sensitize a range of cancer cell types to chemotherapy in preclinical models [39]. Extending this logic, it was further demonstrated that fasting can reverse sorafenib resistance in hepatocellular carcinoma models through a p53-dependent metabolic synergism [151,152].

Critical mechanistic refinement of lipid metabolism challenges the misleading assumption that caloric restriction (CR) and KD are interchangeable. It was demonstrated in models for PDAC that a level of approximately 40% caloric restriction already effectively inhibited tumor growth while a ketogenic diet did not [153]. This was despite both interventions lowering blood glucose to a similar extent. The main difference was the degree of lipid availability: CR (but not KD) effectively reduced lipid levels in the interstitial fluid of tumor tissues. It also inhibited the activity of tumor stearoyl-CoA desaturase (SCD1), enforced SCD overexpression, and finally, resulted in resistance to CR. This finding fundamentally shifts our interpretation of KD versus CR beyond an oversimplified and probably naïve “glucose starvation” logic: it appears likely that altered lipid metabolism may be the decisive axis, at least in some tumor contexts, and CR and KD should not be considered as mechanistically identical.

## 5. The Tumor Microenvironment or TME

### 5.1. The Main Cell Types and Players in the TME

Unlike most targeted drugs used in cancer therapy, KD is a systemic intervention that simultaneously alters a broad spectrum of changes in tumor tissues and metabolism, including nutrient availability, endocrine signaling, immune-cell metabolism, stromal-cell behavior, and tumor-cell substrate use. The TME is therefore not a secondary consideration, but one of the principal determinants of whether a ketogenic intervention sensitizes, fails to affect, or even inadvertently supports a tumor to grow and metastasize.

Cancer-associated adipocytes (CAAs) are particularly relevant, as adipocytes are the primary site of lipid storage and are directly affected by the metabolic shift toward ketosis [154,155]. In obesity-associated cancers, including breast, ovarian, pancreatic, and colorectal cancers, adipocytes in the TME undergo phenotypic changes characterized by delipidation, altered adipokine secretion, changes in lipid storage, and enhanced lipolysis [156,157], often mitigated by regulatory proteins such as FABP4 [156]. How this crosstalk operates within the tumor tissues and the TME, and how it affects the crosstalk between tumor cells and adipocytes under ketogenic conditions, remain largely unexplored. However, this interaction may induce or promote typical effects such as fibrosis and desmoplastic reaction [158], and it may even be involved in developing acquired drug resistance [159]. Systemic ketosis increases lipolysis in adipocytes and potentially enhances fatty acid availability to tumor cells. However, the situation is more complex: ketosis also reduces insulin signaling, which normally promotes the secretion of tumor-promoting adipokines like FABP4 and FABP5 [160,161,162]. CAAs are also known to interact with macrophages and support pro-cancer programs [57]. Recently, it was described that CAAs also act as immunomodulators in solid cancers [163].

Cancer-associated fibroblasts (CAFs) are an extremely heterogeneous class of cells activated by the proximity of tumor cells within tumor tissues [164,165], and some subtypes of CAFs are known to engage in extensive metabolic exchange with tumor cells [166,167]. The ‘reverse Warburg’ model also proposes that some CAFs may undergo aerobic glycolysis [15] and secrete lactate, pyruvate, and ketone bodies, which are eventually consumed by oxidative (Warburg) tumor cells.

Tumor-associated macrophages (TAMs) are key regulators of tumor immunity and are linked to the metabolic state. Although the traditional classification of TAMs into M1 versus M2 states now appears oversimplified, we stick to this for our purpose: M1 (anti-tumor) macrophages rely primarily on glycolysis; in contrast, M2 (pro-tumor, immunosuppressive) macrophages depend on fatty acid oxidation and OXPHOS. This is further supported by interactions between TAMs and adipocytes [54] and by adipokines such as FABP4 and FABP5 [168,169,170]. It is now understood that TAMs exhibit mixed transcriptional programs, and that M1- and M2-associated genes can be co-expressed in the same cell. Lipid metabolism likely drives dynamic shifts between M1/M2 macrophage phenotypes by supplying signals and fuel that push them toward either inflammatory or immunosuppressive states [171], an interaction in which CAFs also play a major role. In tumors, enhanced lipid uptake and fatty acid oxidation typically skew TAMs toward long-lived, mainly immunosuppressive (M2), and tumor-promoting programs [172]. Preclinical studies report variable effects of ketogenic diets on TAM polarization [25]. However, paradoxical findings have emerged: in glioblastoma models, the ketogenic diet increased the M2-like TAM phenotype, necessitating combination with inhibition of colony-stimulating factor 1 (CSF1) and SGLT-2 (sodium-dependent glucose transporter 2) to achieve improved therapeutic benefit [173]. This context-dependence requires deeper mechanistic investigation.

TILs (tumor-infiltrating lymphocytes) and CD8+ cytotoxic T lymphocytes: the effects of ketogenic diets on the functions of T cell-mediated anti-tumor immunity have emerged as a major focus. CD8+ cytotoxic T lymphocytes easily undergo metabolic reprogramming toward aerobic glycolysis, especially upon metabolic activation to support effector function. Memory T cells can also shift toward fatty acid oxidation (FAO) instead of OXPHOS for long-term persistence. Essentially, FAO represents a variant of oxidative phosphorylation focused on fatty acids [174,175] instead of glucose. Tumor-infiltrating CD8+ T cells frequently become exhausted from the effects of aberrant lipid accumulation. CD8+ TILs upregulate CD36 expression and begin to accumulate lipids, leading to lipid peroxidation and ferroptosis, which ultimately eliminates them. High CD36 expression also correlates with poor outcomes in melanoma, colorectal, and pancreatic cancers.

The impact of ketogenic diets on CD8+ function appears predominantly positive, at least in preclinical models. For example, it was demonstrated that the ketogenic diet induced growth retardation of tumor cells in a T cell-dependent fashion [106], with β-hydroxybutyrate supplementation favoring expansion of CXCR3+ T cells. A very recent paper outlined the full mechanistic account of BHB’s enhancement of CAR-T cell anti-tumor function across multiple preclinical cancer models [125], expanding an earlier report [124]. In both publications, BHB was shown to strongly support the Krebs cycle (citric acid cycle, TCA cycle) in CAR-T cells, promoting OXPHOS, proliferation, and cytokine production in T cells. A new clinical trial (NCT06610344) is now enrolling patients with non-Hodgkin lymphoma at Penn Medicine [125].

Myeloid-derived suppressor cells (MDSCs) represent a heterogeneous population suppressing anti-tumor immunity. MDSC metabolism involves lipid uptake and FAO. The effects of KD, CR, and fasting on MDSCs remain almost completely unstudied. This represents a significant gap given their importance in tumor immune evasion.

Synergism of KD with Immunotherapy: Murphy et al. (2024) [27] reported that optimized cyclic ketogenic scheduling with ketone augmentation improved responses to anti-PD-1 and anti-CTLA-4 in prostate cancer models, with simultaneous changes observed in tumor-cell intrinsic immune programs and altered immune infiltration [27]. Candidate mechanisms for this observation include epigenetic remodeling (HDAC inhibition combined with KbHb), altered lactate production and secretion, and increased TME acidity. Other possible players include shifts in T cell differentiation (the spectrum between M1 vs. M2, see above) and changes in myeloid polarization. The putative synergism of fasting and/or KD with immunotherapy—specifically, with ICIs—was reported first in 2021 [106]. This study reported that either a ketogenic diet or BHB supplementation could restore responsiveness to PD-1 blockade and ICIs. The study was performed in resistant mouse tumor models. As a mechanism, they describe T cell-dependent interactions via GPR109A signaling.

### 5.2. The Metabolic Milieu of the Tumor Microenvironment

The TME is defined not only by its cellular composition, but also by its biochemical landscape: the concentrations and fluxes of metabolites within the TME collectively shape the behavior of all resident cells.

Glucose and Glutamine as Dual Anabolic Substrates: cancer cells depend on two principal substrates for substrate-level phosphorylation and biosynthesis: glucose and glutamine. The metabolism of both is orchestrated to produce acetyl-CoA and NADPH for fatty acid synthesis. They simultaneously fuel the production of nucleic acids and amino acids. Cancer cells that can effectively convert these substrates into biomass instead of just energy can outcompete surrounding cells. This can include less advanced tumor cell clones as well as tumor-infiltrating immune cells, which compete for limited nutrients and create a nutrient-depleted microenvironment that suppresses anti-tumor immunity.

Oncometabolites as Regulatory Molecules: cancer cells have effectively learned to exploit metabolic byproducts not only as “food,” but also as signaling molecules. Lactate, which accumulates to 10–40 mM in solid tumors, drives extracellular acidification, thereby impairing CD8^+^ T cell and NK cell function. At the same time, lactate promotes immunosuppressive T-regulatory cells (Tregs), MDSC, and M2 macrophage phenotypes [176]. A recent landmark discovery is histone lysine lactylation (abbreviation “Kla”) particularly at lysine K18 on Histone H3 (H3K18 la). This directly links lactate levels to epigenetic upregulation of target genes, including immune checkpoint molecules such as PD-L1. This provides a new mechanism for metabolic immune evasion. Succinate, for example, accumulates in tumor cells due to succinate dehydrogenase (SDH) mutations or hypoxia within tumor tissues, and stabilizes HIF-1α under normoxic conditions (pseudohypoxia). Succinate also inhibits epigenetic enzymes that depend on α-ketoglutarate (α-KG), leading to DNA/histone hypermethylation. This includes primarily the Fe(II) and α-KG-dependent dioxygenase superfamily. When α-KG levels drop or when competitive inhibitors such as succinate, fumarate, or 2-hydroxyglutarate accumulate, these enzymes lose activity, resulting in DNA and histone hypermethylation. Succinate also signals through Succinate Receptor 1 (SUCNR1), promoting pro-tumorigenic inflammation. Together, lactate and succinate have truly versatile roles, serving as energy sources, immune modulators, epigenetic regulators, and drivers of therapy resistance in cancer cells. In models for colorectal cancer, ketogenic diet-associated microbiome remodeling was shown to lower the levels of methylmalonic acid produced, a known oncometabolite that promotes Rap1-MAPK/ERK-dependent M2-like macrophage polarization. Methylmalonic acid levels correlate with poor patient outcome, and illustrate another route by which a patient’s diet may shape tumor immunity indirectly rather than through ketone body actions alone [177].

Metabolic Heterogeneity: Tumor metabolism is not uniform. Hypoxic cells in the core of tumor tissues, especially deep inside “tumor islands,” largely rely on glycolysis and export lactate via solute carrier family 16 member 4 (MCT4, or monocarboxylic acid transporter 4). In contrast, well-oxygenated peripheral cells near blood vessels within the tumor tissue take up secreted lactate via the transporter MCT1 and use it for oxidative phosphorylation [178,179]. This represents the primary intratumoral lactate shuttle. This metabolic symbiosis optimizes nutrient use throughout the tumor tissue—including “remote” hypoxic areas—and complicates therapeutic targeting. However, the dual inhibition of MCT1 and MCT4 appears necessary to effectively disrupt lactate exchange [180].

Hypoxia-Driven Paracrine Angiogenesis. Hypoxia activates the expression and half-life of hypoxia-inducible factor 1 alpha (HIF-1α), a transcription regulator or modulator that drives glycolysis, VEGF-mediated angiogenesis, and secretion of paracrine factors by CAFs (transforming growth factor beta TGF-β, interleukin 6 IL-6, and Stromal Cell-Derived Factor 1 SDF1/CXCL12) that together promote tumor growth and angiogenesis, and also recruit immunosuppressive cells into the tumor tissue [181] and promote expression and function of PD-L1 [182]. They simultaneously promote epithelial-to-mesenchymal transition (EMT), or the partial EMT version [183,184,185,186] that appears to be prominent in squamous cell carcinomas. Hypoxia, lactate, and succinate are mechanistically interlinked in a self-reinforcing circuit that creates a profoundly immunosuppressive milieu.

Systemic Metabolic Influences: Insulin, Obesity, and Type 2 Diabetes. The metabolic milieu of the TME is also shaped by the host’s systemic metabolic state. Hyperinsulinemia (type 2 diabetes) activates the PI3K/AKT/mTOR axis in cancer cells, stimulating glucose uptake, lipogenesis, and proliferation. In this fashion, cancer cells may be encouraged to use insulin-independent mechanisms of glucose uptake, and/or cancer cells may not become insulin resistant. Chronic inflammation is associated with obesity and driven by pro-inflammatory cytokines such as TNF-α [187], IL-6 [188], and leptin [189]. They are secreted in part by hypertrophic adipocytes and in part by CAFs (especially inflammatory, or iCAFs). These factors, derived from the TME and stroma, strongly promote tumor growth and immunosuppression. The systemic excess of glucose, glutamine, and fatty acids in obese/diabetic patients provides abundant substrates for rapidly proliferating tumor cells. Consistent with the observation that the host state represents a substantial part of the treatment mechanism, the delayed tumor progression by ketogenic diet was observed preferentially in obese GEMM models for pancreatic cancer, but not in slim mice. This was quite independent of weight loss, indicating that a baseline metabolic phenotype may determine whether the same diet is protective, neutral, or potentially adverse [190].

A summary—How Ketogenic Therapy Reshapes the Milieu: We have seen that ketogenic metabolic therapy targets multiple components of this metabolic landscape simultaneously:

• Lower glucose translates into lower lactate levels: reduced glycolytic flux is expected to attenuate intratumoral lactate accumulation, acidification, and lactylation-driven immune evasion.
• Lower insulin: reduced insulin and IGF-1 levels directly attenuate PI3K/AKT/mTOR signaling and secretion of adipokines that promote tumor growth.
• Elevated ketone bodies: the central metabolite BHB inhibits histone deacetylases (HDACs), activates HCAR2, and promotes AMPK-mediated degradation of PD-L1, and BHB also functions as an endogenous immune checkpoint modulator.
• Elevated systemic free fatty acids (FFAs): This is perhaps the most complex and underexplored consequence. Enhanced lipolysis increases circulating free fatty acid (FFA) levels, which may provide an alternative fuel source, especially for metabolically flexible cancer cells, and promote immunosuppressive phenotypes in TAMs and MDSCs through increased FAO. FFAs may also interact with the gut microbiome, especially via stearate [129], and control colorectal cancer progression. Elevated levels of polyunsaturated fatty acids (PUFAs) may induce ferroptosis in some cancer cells that have only limited antioxidant capacity [191,192]. There are also other reports that KD may promote ferroptosis in cancers [126]. In contrast, some recent studies report conflicting evidence. For example, it was demonstrated that ketogenic diet-induced transcriptional activation via the BACH1 transcription factor can promote metastasis in certain breast cancer models [120], which underscores that the net effect is highly context-dependent.

The dual nature of these metabolic shifts, particularly the role of FFAs and PUFAs, warrants careful mechanistic investigation and tumor-specific metabolic profiling to identify patients most likely to benefit from ketogenic metabolic therapy.

### 5.3. Integrating TME Complexity in Model Systems

The preceding survey reveals profound complexity: 1) Cancer cells vary in ketolytic capacity. 2) Adipocytes may provide fatty acids that support or stress cancer cells. 3) CAFs engage in metabolic symbiosis that may be disrupted or redirected. 4) TAMs may shift toward anti-tumor or immunosuppressive phenotypes. Finally, 5) T cells may be enhanced or impaired depending on their differentiation state. This complexity presents both a challenge and an opportunity. Simple model systems cannot capture emergent behaviors from multicellular metabolic networks. Systematic investigation using increasingly complex models, from monocultures to co-cultures to organoids to in vivo models, can progressively build understanding while testing specific mechanistic hypotheses.

## 6. Experimental Model Systems

Experimental work on KD and CR, glucose restriction, FMD, and exogenous ketone delivery (Box 1) should not be interpreted as implying that these different interventions are metabolically equivalent. The model systems surveyed below are also the testbed for this review’s central, falsifiable predictions (Figure 5 and Figure 4). The available literature shows that the choice of a model determines which layer of biology is being interrogated: this can include direct ketolytic competence of tumor cells versus stromal contributions. Since metabolic processes are addressed, we need to take necessary precautions in model systems, which are sometimes neglected or ignored: across in vitro systems, three methodological variables routinely confound the interpretation of oncometabolic experiments and deserve explicit methodological reporting. First, serum composition: standard fetal calf serum contains uncontrolled amounts of insulin, IGF-1, glucose, and lipid species that can dominate over any experimental manipulation of ketone body concentration. Second, antibiotics such as aminoglycosides and β-lactams (penicillin) used routinely inhibit mitochondrial ribosomes and can induce a Warburg-like metabolic shift independent of any dietary intervention. Third, oxygen tension: atmospheric incubator conditions (21% O_2_) are non-physiological; the physiological O_2_ pressure in tissues ranges from ~5% to <3% in tumor hypoxic zones, and HIF-1α stabilization, together with MCT4-driven lactate export, is strictly O_2_-dependent. The pros and cons of the most relevant in vitro and in vivo model systems are summarized in Figure 7.

### 6.1. Two-Dimensional Monoculture Systems

Two-dimensional (2D) cancer cell cultures remain indispensable for mechanistic work because substrate concentrations can be precisely defined, genetic perturbations (siRNA; CRISPR-Cas9 gene editing; retro- or lentiviral gene transfer) are often straightforward to implement, and high-throughput (or high-content) phenotyping is feasible. Two-dimensional monolayer cultures are the most appropriate systems for quickly and cost-effectively determining whether a tumor cell can import and metabolize ketone bodies, whether BHB acts primarily as a fuel or as a signaling metabolite, and whether low-glucose states (or media) may modify responses to chemotherapy, radiotherapy, or targeted agents. How physiologically relevant these 2D findings are is another question.

At the same time, 2D monocultures are also where the field’s intrinsic contradictions become most visible. In several breast cancer models, physiological BHB altered measurements of oxygen consumption rate (OCR) and extracellular acidification rate (ECAR) (e.g., using the Seahorse Extracellular Flux Analyzer, Agilent Inc., Palo Alto, CA, USA) and other metabolic readouts, but did not change proliferation or sensitivity to chemotherapy or radiation [17]. Similarly, glucose deprivation, i.e., lowering glucose levels in the media, effectively reduced growth and proliferation in “classic” breast cancer cell lines such as MCF-7 and T47D. However, adding BHB did not rescue that phenotype or add any major pathway-level effects [193]. In glioma cell lines, BHB rescued normal, non-transformed neurons from glucose deprivation, but failed to protect malignant glioma cells, despite detectable ketolytic enzyme activity. This indicates that enzyme expression alone does not guarantee the meaningful use of ketone bodies by different cells under stress conditions [194]. They demonstrate that any increase in circulating ketones does not automatically translate into a tumor-relevant phenotype, at least not in vitro.

Other studies in 2D cultures show the opposite: clear context-dependent growth support, stress induction, or therapy resistance. For example, one study reported line-specific and highly variable effects of BHB on viability, colony formation, ROS, and the transcriptional programs in breast cancer cells [195]. Another study showed that human breast cancer cells exposed to BHB displayed consistently increased viability, proliferation, migration, and resistance to 5-fluorouracil (5-FU) [196]. In a glucose-restricted model using A549 lung cancer cells, long-term BHB exposure promoted apoptosis, increased ROS accumulation, increased mitochondrial dysfunction, and reduced the expression of stemness-associated markers. This display of metabolic plasticity can also be exploited therapeutically in selected settings [197]. Rodrigues et al. described a “β-hydroxybutyrate paradox” in mouse mammary tumors: at relatively low concentrations, BHB was consumed as an oxidative fuel, accelerating tumor growth [198], and did not measurably increase histone H3 acetylation. Taken together, these data argue that 2D systems are not always very useful for proving simplified paradigms like “ketones are good” or “ketones are bad”; however, they may be suitable for mapping which cell lines and tumor types are likely indifferent, vulnerable, or opportunistic to ketones (and therefore likely to KD).

Some positive observations indicate that monoculture systems can still be valuable. First, ketolytic enzyme profiling in simplified monocultures can be readily used as a stratification tool. For example, in malignant gliomas, low expression of key ketolytic enzymes like 3-Oxoacid CoA-Transferase 1 (OXCT1) and 3-Hydroxybutyrate Dehydrogenase 1 (BDH1) relative to glycolytic enzymes suggests that only a subset of tumors might be genuinely sensitive to ketosis [63]. This may also apply to other cancer models (cell lines), and it is likely to indicate that low expression of rate-limiting ketolytic enzymes often predict stronger ketogenic responses in vitro and in vivo [199]. Similar studies have shown that BHB increases cytosolic acetyl-CoA production via a non-canonical route that depends on Acetoacetyl-CoA Synthetase (AACS) expression and activity. This likely means that carbon atoms provided by the ketone carbon can be utilized for anabolic metabolism even when glucose remains available [200]. This mechanistic refinement is important because it explains why some tumors may use ketones for biosynthesis, while others may not, and why, instead, they preferentially oxidize ketones for energy in the mitochondria.

The major weakness of 2D work is that standard media can introduce substantial metabolic artifacts compared to human tissues. For this reason, human-like cell culture media like Plasmax have been developed [201]. These approaches show that supraphysiological levels of pyruvate, glucose, amino acids, and imbalances in micronutrient levels can severely distort cell behavior [202,203]. However, even in such more physiologic media, rapid nutrient depletion can push cells into unintended starvation within 48 h unless the media are changed frequently [201]. Accordingly, the most informative 2D experiments performed in 2D monolayer conditions (or classic cell culture) for any questions related to KD or CR are not simple “let us put some BHB in DMEM” assays. The more informative assays at least aim for (and still do not necessarily fully achieve) glucose-controlled, physiologic medium designs.

### 6.2. Co-Cultures: Tumor-Stroma and Tumor-Immune Cultures

Co-culture systems are the natural next step because they capture nutrient exchange among different cell types (such as tumor cells exposed to CAFs, adipocytes, mesenchymal stem cells, or immune cells) without sacrificing standardization and robustness. Co-culture models are especially relevant to ketogenic research because tumor cells are rarely exposed to ketones in isolation; they encounter them alongside stromal lipids, metabolites, cytokines, and extracellular vesicles.

Naturally, adipocyte-tumor cell co-culture models should be among the strongest and most relevant examples for metabolic research. However, ex vivo culture of adipocytes is extremely challenging. Adipocytes (often called CAA or cancer-associated adipocytes) actively transfer FFAs to breast cancer cells and promote fatty-acid oxidation (FAO), cell proliferation, and tumor cell motility and migration [204]. As expected, stronger effects are observed in co-culture with adipocytes derived from obese patients [204]. Adipocytes derived from breast tissue, when co-cultured with tumor cells, can supply BHB directly to breast cancer cells expressing monocarboxylate transporter 2 (MCT2, also known as solute carrier family 16 member 7; SLC16A7). BHB supplementation via contact with adipocytes increases H3K9 histone acetylation and induces the expression of tumor-promoting genes in breast cancer cells [205]. These studies show that the most relevant ketone source may be of stromal and local origin, but not only systemic. Nevertheless, some work on pancreatic cancer illustrates that both systemic and local ketone supply may have comparable effects. Supplementing cell culture media with ketone bodies reduced glycolytic flux, glutamine uptake, ATP levels, and survival in PDAC cells and cell lines grown in 2D, and attenuated cachexia-related effects in both co-culture settings and orthotopic mouse models [206].

For the co-culture of tumor cells with CAFs, direct evidence on ketosis remains comparatively sparse, but at least the conceptual framework and experimental setup is well established. One of these tumor/CAF co-culture models showed that oxidative stress and stromal catabolism generate ketones and other metabolites that can fuel adjacent cancer cells [207]. The “reverse Warburg” model (Pavlides/Lisanti lab) proposes that CAFs, not the tumor cells, may undergo aerobic glycolysis under paracrine signals from adjacent tumor cells [15]. The CAFs then secrete lactate, pyruvate, and ketone bodies that are imported by oxidative tumor cells via MCT1 and used to fuel OXPHOS [16,179]. In effect, the stroma feeds the tumor [15,16,208,209,210]. In addition, CAF-derived exosomes can deliver metabolites and partially reprogram tumor metabolism, especially under nutrient or hypoxic stress [211].

Immune cell co-cultures play a slightly different role and are technically challenging, especially when investigating metabolic questions. Only a few studies exist. Luda et al. demonstrated that ketolysis is a direct determinant of CD8+ T cell effector function, with BHB-derived acetyl-CoA supporting histone acetylation at effector loci. OXCT1-deficient CD8+ T cells showed impaired histone acetylation at effector-gene loci and reduced in vivo tumor control, establishing ketolysis as a direct (not merely correlative) driver of CD8+ effector function [212]. This study is not yet comparable to a mature, defined, and standardized tumor/immune co-culture model system, performed under ketogenic conditions, but it provides a compelling biological rationale for moving beyond epithelial-only systems when the aim is to model immunotherapy-relevant dietary interventions.

### 6.3. Three-Dimensional (3D) Spheroids and Multicellular Tumor Spheroids

Three-dimensional spheroids are the first widely accessible models in which nutrient diffusion, oxygen gradients, and spatial metabolic heterogeneity become intrinsic properties of the system. For ketogenic and glucose-restriction research, that matters because cells in the inner spheroid core versus the outer periphery may face very different growth and nutrient or substrate landscapes. When comparing 2D and 3D tumor-on-chip settings, 3D cultures generally exhibit lower proliferation, altered glucose dependence, glutamine and lactate dynamics, and distinct adaptation to glucose restriction compared with cells grown in 2D monolayer cultures [213]. This may relate to how tumor cells in vitro respond to BHB levels: one study found that BHB increased spheroid size in such 3D cultures and reduced the efficacy of 5-fluorouracil (5-FU). This may indicate that a pro-survival BHB phenotype can persist or become more effective when cells are transferred from flat 2D culture to structured 3D assemblies [196]. Another study connected ketogenesis and BHB with changes in migration, invasion, and metastatic progression in pancreatic cancer. Such dynamic phenotypes are often more visible in 3D than in short-term 2D assays [121]. Spheroids are therefore useful as an intermediate approach, but are often mistaken for organoids (see below). Spheroids, although typically less organized and polarized than organoids, can still determine whether, for example, direct BHB effects persist after the acquisition of 3D multicellular aggregates and architecture. Spheroids are quickly generated and are suitable for assessing whether low-glucose responses are diffusion-limited rather than intrinsic or whether stromal support (by co-cultured CAFs) creates protected metabolic niches. Their main limitation is that gradients remain poorly controlled and are difficult to measure compared with microfluidic platforms.

### 6.4. Patient-Derived Tumor Organoids (PDOs) and Organoid-Based Immune Models

Organoids, especially patient-derived organoids (PDOs), bring patient specificity, more genetic fidelity, and the potential to form tissue-like structures, such as epithelial architecture, within experimental reach. PDOs are particularly attractive for ketogenic research because they can test whether a mechanistic effect observed in a cell line generalizes to clinically relevant tumor tissue.

The strongest proof of principle in the field to date showed that BHB supplementation in organoids suppresses colorectal cancer growth through an interaction between Hydroxycarboxylic Acid Receptor 2 (HCAR2) and the HOP homeobox protein (HOPX). The study demonstrated that this is active in both organoid systems and in vivo [113]. This study is especially valuable because it treats BHB not merely as a fuel for cancer cells, but also as a signaling metabolite whose effects depend on the receptor (HCAR2) and the transcriptional context (HOPX). There are also complementary organoid-linked perspectives. One study investigated the impact of short-term starvation and interventions that mimicked fasting in organoid models [112] of breast cancer [40]. In this setting, complementation of media with BHB significantly enhanced endocrine therapy responses in hormone receptor-positive breast cancer, including in organoid experiments. This effect of BHB was observed, although the most relevant biology was driven more likely by systemic growth factor restriction than by utilization of ketones per se [40]. A ketogenic setting is, however, not uniformly protective in the intestine [214]. In genetically susceptible mouse models, a ketogenic diet accelerated small-intestinal adenoma formation and shortened the survival. Genetic manipulation of ketone body production or ketone utilization in these mice did not reproduce this phenotype. Instead, PPAR-dependent lipid oxidation and CPT1A-mediated fatty acid utilization likely promoted intestinal stem cell expansion and tumorigenesis [214]. The effect was therefore primarily attributed to the lipid and fatty acid oxidation component of the diet rather than to ketone bodies themselves. This supports a model in which dietary ketosis cannot be reduced to the actions of just BHB or AcAc. However, this is a tumor-initiation model in particularly susceptible mice, and does not necessarily demonstrate that KD accelerates the progression of established human epithelial cancers.

Organoid-immune co-cultures remain a promising yet largely hypothetical approach; they are technically challenging and do not seem sufficiently mature to investigate fundamental questions about cancer metabolism and its impact on immunotherapy. While publications based solely on immune cells suggest such functional connections, direct studies of organoid-immune co-culture systems under ketogenic conditions remain scarce. This should be framed as a future aspect and as an opportunity, but cannot be presented here as a mature development.

### 6.5. Microfluidic Platforms and Tumor-on-Chip Systems

Microfluidic systems are among the most conceptually aligned platforms for ketogenic and caloric-restriction research because they can impose defined gradients, timed substrate switches, and spatially resolved drug exposure. In other words, they allow investigators to model the fact that dietary interventions do not change tumor metabolism uniformly across space or time.

A simple microfluidic platform was developed that enabled real-time profiling of oxygen and glucose gradients in cell cultures. The same microfluidic devices that provided a tumor-like chamber for long-term cell culture also allowed measurement of viability, proliferation, ROS, and apoptosis [215]. This tool was constantly further developed [216]. In several follow-up studies, the same platform concept or family of devices was used to examine how tumor cells may respond to environmental gradients and to map their metabolic vulnerabilities when exposed to inhibitors of glycolysis, fatty acid oxidation, and oxidative phosphorylation. Each study was performed under controlled nutrient asymmetry [217,218]. These papers could be considered pioneering studies that demonstrated that the question is not simply whether a tumor can use ketones, but rather which tumor regions may become vulnerable or protected when nutrients are restricted and then redistributed.

A few additional studies show how 2D and 3D chip-based organization changes metabolite consumption patterns [213,219]. Carneiro et al. discussed in a recent review article how the use of tumor-on-chip platforms, as part of the emerging landscape of single-cell and patient-derived cancer microfluidics, will (hopefully) integrate aspects that simpler, static co-culture models outlined above were unable to address, such as immune compartments, biosensing, and physiologically relevant tissue organization [219]. PI3K inhibitors such as Alpelisib (FDA-approved for PIK3CA-mutant HR+/HER2- breast cancer) and Inavolisib are clinically used anticancer drugs whose efficacy is limited by a systemic glucose-insulin feedback loop: PI3K blockade causes hyperglycemia, which raises insulin, which in turn reactivates tumor PI3K signaling. Hopkins and colleagues showed in mouse models [32] that suppressing this feedback mechanism, either pharmacologically or with a ketogenic diet, restores the efficacy of PI3K inhibitors. This provides a direct rationale for KD as a metabolic adjuvant to an approved targeted therapy [32].

### 6.6. Precision-Cut Tumor Slices and Explant Cultures

Among the most translational ex vivo systems, precision-cut tumor slices and related tumor explant cultures remain underappreciated in ketogenic research. Such models have a key advantage: they preserve the native tumor architecture (at least for several days), do not massively change the original stromal composition, and, in some settings, can even include or retain clinically relevant immune populations. At the same time, these models still allow controlled short-term interventions and are relatively robust and reproducible.

One hallmark study showed that precision-cut slices can preserve complex tumor biology in vitro, provided they are handled with appropriate support materials and maintained with sufficient continuous oxygenation. The authors warn that temporal and spatial stress responses must be accounted for, as they may affect interpretation [220]. An organotypic human PDAC slice model has been shown to preserve stromal complexity and metabolic activity for several days [221]. Another standardized tumor slice culture model was presented as a biologic surrogate of human cancer [222]. Yet another study demonstrated that the responses of NSCLC explant cultures to cisplatin correlated well with patient survival. Such positive observations make this class of models particularly attractive for therapy-modulation studies [223]. These studies demonstrate that both the original tissue architecture or histology and viable immune populations can persist for sufficient periods to enable ex vivo chemosensitivity assays and functional drug testing [224,225]. Finally, one study aimed to bridge the technological gap between slice culture and microfluidics. The authors created a platform for multiplexed drug testing on intact tumor slices [226]. This “slice-on-chip” approach may be one of the more relevant future chemosensitivity platforms for personalized medicine and early-stage drug discovery. It can also be used to conduct experiments involving ketogenic, low-glucose, or ketone-ester exposure. This platform combines native tissue complexity with controlled delivery, which is a first in the field.

However, this should all be considered carefully, as there are still significant gaps in available technologies and in physiological understanding. The slice literature may be relatively strong methodologically, but almost none of it has yet been applied using a rigorously designed ketogenic medium (as described above). Tissue or tumor slices are already a mature translational platform, but they have not been widely used to address metabolic and nutritional questions.

### 6.7. In Vivo Dietary-Intervention Models

Animal models remain indispensably relevant and critical for metabolic research. Only whole organisms can faithfully reproduce the dynamics of hepatic ketogenesis, insulin, and IGF-1. Only animal models mimic fully functional adipose lipolysis, microbiome contributions, and systemic immune adaptation. The most frequently used mouse models employ “classic” human tumor cell lines as xenografts in immunodeficient “nude mouse” hosts.

In a prior xenograft study, a ketogenic diet enhanced oxidative stress and radio-chemo responses in lung cancer xenografts [227], but these models lacked immune cells. More recent studies have reported enhanced immunity targeting glioma cells in a mouse model under KD conditions. They observed more immune cells, and more tumor-reactive CD8+ and NK activity, and reduced inhibitory receptor expression under the conditions of KD [26]. Then, even more recently, immunocompetent mouse models were used to demonstrate that KD and ketone bodies can enhance the efficacy of PD-1 blockade [106]. This logic was further extended to checkpoint-resistant prostate cancer: it was demonstrated that cyclic KD and/or BHB supplementation can reshape both tumor-intrinsic and immune features of response [27]. Furthermore, dietary restrictions improved anti-tumor immunity in mouse models. Oxidization of β-Hydroxybutyrate (βOHB) helped to maintain a more functional CD8+ T cell state in tumor-infiltrating cells within the TME [228].

Several animal studies strongly warn against treating KD, CR, and exogenous ketone delivery as equivalent and interchangeable interventions. Some animal studies have shown that calorie restriction, but not a ketogenic diet, suppresses tumor growth in their models. They suggest that the decisive variables were reduced lipid availability and impaired lipid desaturation (based on Stearoyl-CoA Desaturase, or SCD1, activity), rather than lower glucose alone [229]. Another investigation found that a non-calorie-restricted KD may have effectively raised blood ketones but did not lower glucose levels, nor did it improve survival in a glioma model [194]. Several reports support the basic findings described in the recent literature on FMD in human patients. Here, the major biological effects likely arise from coordinated changes in the secretion and circulation of growth factors, glucose, and amino acids, and from altered immune states, but not simply from ketone exposure [40,112,230,231]. Exogenous exposure to ketones and ketone esters represents yet another category that has to be taken more carefully: anti-tumor effects in murine models were achieved in some models even without classical dietary carbohydrate restriction, just by supplementation with ketones [232,233]. In contrast, others find that ketones or ketogenic diets can even support and promote tumor progression, and that this is strongly dependent on tissue context and lipid metabolism [121,198,214].

The practical implication is that in vivo work should ideally be used to test model-specific hypotheses that were generated upstream, but it should not be used simply to repeat a generic ketogenic intervention across multiple tumor types. Whole-animal studies are most informative when tumor ketolytic capacity, lipid dependence, and immune context are already characterized, and when circulating biomarkers such as BHB, glucose, insulin, and lipid species are measured combined with tumor outcomes.

## 7. Experimental and Analytical Frameworks

### 7.1. Simulating Ketogenic Conditions In Vitro

Most in vitro “ketogenic” experiments only manipulate glucose and add BHB, while leaving all other components of the metabolic milieu—insulin, glutamine, fatty acids, lactate, and growth factors—at non-physiological levels. For mechanistic work in oncometabolism, culture conditions should instead approximate the systemic milieu of either a fed, insulin-replete state or therapeutic ketosis.

In a healthy human, typical circulating values are: glucose ~5 mM (postprandial 7–8 mM), insulin ~10 mIU/L (70 pmol/L), glutamine 2–4 mM, BHB 0.05–0.25 mM, AcAc 0.01–0.16 mM, and lactate ~0.5–1 mM in healthy people (∼2 mM in type 2 diabetes). Mixed plasma FFAs should be in the 0.3–0.6 mM range; most of them are albumin-bound. Under strict ketogenic or therapeutic ketosis conditions, glucose levels fall to ~2.5–3.5 mM, and insulin is strongly suppressed to ~2–5 mIU/L (14–35 pmol/L). In contrast, glutamine remains around 2 mM, while BHB rises to 2–6 mM, AcAc to 0.5–2 mM, and lactate usually drops to <1 mM. Plasma FFA concentrations concurrently rise, especially with prolonged fasting or very-low-carbohydrate intake, and may reach ~0.8–1.5 mM total FFAs, while they are still largely albumin-bound. To minimize non-physiological lipotoxicity in oncometabolic culture assays, free fatty acids should be complexed to BSA at molar FA:BSA ratios not exceeding 3:1.

Acute BHB exposure (hours) elicits rapid signaling effects, but to reach peak effects, multi-day exposure is required for transcriptional and organelle-level adaptations. A single endpoint after ‘72 h in BHB’ is insufficient, and conflates acute signaling with adaptation. Best-practice conditions would be: 1) predefine exposure times (6–24 h acute; 3–7 days chronic), 2) collect time-resolved readouts, and 3) report the passage number and adaptation strategy. Figure 3 captures the dynamics of metabolic changes and the need for multiple time points when using diverse model systems, compared to clinical trials: in vitro and clinical timelines span a broad range of hours, days, weeks, and months. A single endpoint at any given time cannot possibly capture the full response. Figure 3 also highlights that early effects may not persist, and that resistance mechanisms (and their consequences) may emerge over months. It is therefore critical that both experimental in vitro studies and clinical trials match the physiological timescale of the biological question being asked.

As mentioned previously, serum is a major hidden variable in the experimental setup, contributing glucose, lipids, growth factors, and hormones. When the question is ‘what does ketosis do to tumor metabolism,’ investigators often need to decouple ketone availability from confounding growth factor stimulation. This can be done by using reduced serum concentrations or defined supplements, and, more explicitly, by controlling insulin/IGF signaling. Routine use of penicillin/streptomycin or gentamicin in cell culture can also be problematic, as it introduces a critical confounding factor in oncometabolic studies. Consequently, they can upregulate glycolytic enzymes such as PKM2 and LDHA, effectively inducing a Warburg-like phenotype independent of any experimental treatment. Oncometabolic studies must therefore be conducted in antibiotic-free media to ensure that observed metabolic phenotypes reflect true biological responses rather than antibiotic-induced artefacts.

### 7.2. Analytical Technologies

Lipidomics is particularly informative in ketogenic contexts because ketone availability and glucose restriction jointly influence acetyl-CoA supply, NADH/NAD+ balance, and lipogenic versus oxidative flux. Shotgun lipidomics provides broad coverage, while targeted LC-MS/MS improves quantitative accuracy for defined lipid classes. Stable isotope tracing such as ^13^C can distinguish whether ketone bodies act as oxidizable substrates (feeding through the TCA cycle), as redox modulators, or as signaling metabolites [234]. Isotope tracing was used in a study that introduced a novel method the authors called “13C-SpaceM,” used here for spatial single-cell isotope tracing of de novo lipogenesis [235]. The method uses imaging mass spectrometry for the spatially resolved detection of a ^13^C label (derived from ^13^C 6-glucose as input), which is then incorporated into esterified fatty acids, combined with microscopy and computational methods for data integration and analysis. This represents a novel and attractive approach for understanding how ketogenic states differentially affect tumor core versus margins.

Seahorse extracellular flux analysis is now widely used in ketogenic studies. It can specifically be used to detect shifts between oxidative and glycolytic phenotypes. For ketogenic studies, flux analysis can test whether cells can oxidize BHB by measuring OCR changes upon ketone supplementation. Imaging modalities reading out metabolism in space—hyperspectral redox imaging, FLIM of NAD(P)H/FAD, mass spectrometry imaging—are more informative than bulk averages for 3D systems.

Functional assays remain essential because metabolic rewiring does not necessarily translate to growth inhibition. Proliferation under glucose-restricted and ketone-supplemented conditions, clonogenic assays for long-term survival, migration/invasion in 3D, and drug sensitivity testing should all be embedded in a defined metabolic context and paired with metabolic verification.

## 8. Knowledge Gaps and Future Directions

### 8.1. Unresolved Mechanistic Questions

Why do some tumors (and patients) respond to KD or CR, while others do not? A key variable appears to be ketolytic capacity [63,128,199]. This study provides a striking example: lung tumor-initiating cells (TICs) were shown to switch to ketone utilization under glucose limitation, representing potential targets for chemotherapy [147]. However, prolonged KD and TIC reprogramming also creates targetable dependencies, for example, on the activity of the MCT1/CD147 system [178] or FASN activity.

Clinical ketogenic interventions produce complex and context-dependent systemic changes that affect tumor cells, the TME, the immune system, and host metabolism. Dissecting the contribution of each intervention and each confounding factor remains challenging. The finding that the anti-inflammatory effects of KD require a minimum of >12 weeks duration suggests that chronic immune modulation may be more important than acute metabolic effects for some outcomes.

Optimal timing of KD, especially relative to standard therapies, remains largely undefined [236]. Do tumors adapt to sustained ketogenic conditions, and through what mechanisms? Identifying resistance mechanisms could reveal vulnerabilities in combination approaches. Reference [27] highlights that cyclic scheduling of KD and/or CR may retain long-lasting anti-tumor effects while mitigating the potentially adverse effects of continuous exposure, e.g., to BHB, as mentioned above.

### 8.2. TME-Specific Gaps

Despite the central role of CAFs in tumor metabolism and metabolite exchange, there is remarkably little direct work related to testing CAF-specific responses under defined ketogenic conditions. The role of stromal catabolic phenotypes and ketone body-related pathways is discussed in Ref. [237], largely as seen through the lens of earlier “reverse Warburg” paradigms [15,16,179]. To investigate this in greater detail, more rigorous CAF-tumor co-culture experiments under controlled glucose/ketone/fatty-acid compositions would be necessary—ideally with metabolic flux readouts [234] and CAF subtype resolution, as demonstrated in these examples from head and neck cancer [238,239]—but such studies remain scarce.

Adipocyte-tumor crosstalk under ketogenic conditions also remains largely unexplored. The interaction of KD with MDSCs is virtually unstudied. Similarly, NK cells and dendritic cells await systematic investigation under KD and control diet (CD) conditions. The spatial organization of metabolic niches within tumors under ketogenic conditions presents a major opportunity, but as of yet, only a few examples have been published [235]. Recent work in colorectal cancer also indicates strong coupling between KD and the gut microbiome. For example, KD suppressed tumor burden in a humanized mouse model, supplemented with a human microbiome. Mechanistically, this likely occurred via increased stearate levels in the gut [129]. This suggests that incorporating microbial considerations and adjusting metabolite readouts accordingly could be decisive for more precise insights.

Complex co-cultures that contain cancer cells, combined with CAFs, adipocytes, or immune cells (Section 6)—ideally cultured under strictly controlled ketogenic conditions—still represent a technical challenge, but they may have a high potential impact. Finally, the consistent use of standardized ‘ketogenic medium’ formulations [202,203], introduced in Section 6, should be validated across many laboratories to accelerate reproducible research. As of yet, only a few laboratories use such media formulations. Additionally, for future studies, diet formulations should be rigorously optimized, standardized, ideally informed by the mechanisms under investigation. This would include characterizing the origin of fats and fatty acids in the experimental system (cells, organoids, tissues, etc.), the protein quality, and the corresponding source of micronutrients.

CAFs within tumors are often senescent and show a phenotype for which the term SASP was coined: senescence-associated secretory phenotype (SASP). In CAFs undergoing SASP, a pro-inflammatory, pro-tumorigenic mixture of growth factors, chemokines, and cytokines is secreted that promotes therapy resistance and metastasis [238,239]. Because BHB modulates SIRT1, AMPK, mTOR, and p53 expression and activities, KD might act partly by suppressing the SASP rather than by targeting tumor cells directly. As of yet, the available experimental evidence is sparse and points in both directions [240,241]. In mice, two different types of KDs induced p53-dependent cellular senescence in heart and kidney. Other studies with human probands showed elevated SASP markers in serum and also in plasma from patients enrolled in a 6-month KD trial. The pronounced senescence was cleared by a senolytic agent and prevented by an intermittent KD [235]. Whether ketogenic conditions suppress stromal SASP within the tumor tissue, while simultaneously raising it systemically in normal tissue is unresolved. The possible answer likely depends directly on whether continuous or cyclic protocols are preferable (Section 8.1). Here, we could suggest a simple testable approach: (1) induce senescence in defined CAF subtypes, (2) expose them to standardized ketogenic medium, and (3) quantify SASP output; finally, (4) measure senescence and SASP markers in tumor and matched normal tissue in clinical KD studies.

Epigenetic memory. BHB inhibits class I histone deacetylases [135] and serves as the substrate for histone lysine β-hydroxybutyrylation [19,20]; both alter chromatin state and gene expression. Whether these marks outlast the diet is unknown. If they do, a bounded “metabolic priming” window might deliver durable benefit, and brief exposure could resensitize—or desensitize—therapy-resistant cells by erasing resistance-associated marks. The same question applies to tumor cell plasticity [242], dormancy [243], and lineage transitions such as partial EMT [184]. This leads us to another testable approach: (1) brief versus extended ketogenic exposure is combined with single-cell ATAC-seq and lineage tracing, (2) asking which loci retain specific epigenetic marks, (3) in which cells these marks are prominent, and (4) for how long they are retained after withdrawal.

Dormant disseminated tumor cells. Most cancer deaths follow reactivation of disseminated tumor cells that survive initial therapy [243]. These cells are typically quiescent, autophagy-dependent, and oxidatively competent—a phenotype for which ketones are usable fuel. KD could therefore deepen dormancy by reducing proliferative signals such as insulin and IGF-1, extending time to relapse, or shorten it by supplying reactivating cells with an alternative substrate. The two predictions give opposite clinical advice: KD as maintenance therapy after active treatment, versus KD as a hazard in patients with occult disease, particularly when fasting or ketosis is prolonged. Reactivation kinetics have not been tracked under ketogenic conditions, largely because dormant cells are difficult to capture. Finally, we can approach this question with another testable approach: organ-chip systems that sustain dormant tumor cells and permit their reactivation in vitro [244] make this question addressable on a timescale that long-term animal experiments do not allow.

Mode of cell death, not magnitude. Apoptosis, ferroptosis, pyroptosis, necroptosis, and autophagy differ sharply in immunogenicity: some prime anti-tumor immunity, others are immunologically silent or tolerogenic [245]. Several ketogenic effects converge on this choice—altered redox state, membrane lipid composition, which sets the ferroptosis threshold [183,184], and BHB-mediated NLRP3 inhibition [21]. The latter should also restrain pyroptosis. KD promoted tumor ferroptosis in vivo, although in the same model it also induced a relative corticosterone deficiency that accelerated cachexia [123]. Which death pathway is eventually engaged, rather than how much killing occurs, may explain the inconsistent synergy between KD and immunotherapy. Testable approach: one could analyze parallel readouts of lipid peroxidation, gasdermin D cleavage, and immunogenic cell death markers, each under defined ketogenic versus normal conditions; or, they can be investigated in patient tissue before, during, and after therapy.

The membrane lipid code. Membrane lipid composition governs receptor localization, assembly of signaling platforms, drug uptake, immune synapse quality, and sensitivity to ferroptosis. KD alters systemic lipid availability and therefore the fatty acid composition of membranes. However, the corresponding hypothesis that part of the KD effect is membrane-mediated and independent of intracellular ketone metabolism has not been experimentally tested. The fact that lipid-derived signals can effectively suppress T cell anti-tumor function through myeloid intermediaries [170] indicates the magnitude such effects may possibly reach within tumor tissues. Testable approach: One could perform lipidomics of isolated membrane fractions rather than whole-cell lysates under standardized ketogenic media. This should then be combined with super-resolution imaging of receptor clustering within the membrane and supported by functional immune synapse assays.

None of these five central, underinvestigated questions requires new technology development. Each of them only requires that an existing readout is systematically applied under controlled ketogenic conditions and compared to normal media. Naturally, this should be conducted in informative, physiologically relevant models that actually contain the relevant cell types, which are still rare. Together, these five questions define which parameters a mechanistically informative KD study would need to measure, and how far current practice remains from that standard.

### 8.3. Pharmacological Modulators, Ketogenic Mimetics, and Hyperbaric Oxygen (HBOT)

An emerging translational strategy involves pharmacological agents that mimic, enhance, or synergize with the effects of the ketogenic diet, either by pharmacologically inducing ketosis, reducing glucose availability, or targeting the same metabolic pathways modulated by ketones. Conceptually, keto-mimetic pharmacology rests on the broader framework of cancer metabolic reprogramming and nutrient microenvironment constraints [136,137,246,247]. Most importantly, tumors can adapt via alternative substrate use, e.g., using or abusing lactate symbiosis, glutaminolysis [248], serine biosynthesis, and lipogenic rewiring. These mechanisms cooperate, which motivates researchers to explore and test combination strategies, supported by biomarker-guided selection [146,249,250,251,252]. The principal pharmacological strategies that may complement or reinforce ketogenic metabolic interventions, together with metabolic targets and endpoints aligned with the ketogenic diet, the corresponding mechanistic rationale, and representative evidence, are summarized in Table 4. In clinical testing, however, the benefit of these repurposed agents has been modest and inconsistent—recent randomized trials of metformin in cancer, in particular, have been largely negative—so they are best regarded as investigational adjuncts rather than established therapies.

**Table 4:** Ketogenic interventions and metabolic drug targets.

Class/Examples	KD-Aligned Metabolic Endpoint	Mechanistic Anticancer Rationale	Representative Evidence
Metformin/AMPK activators	↓ hepatic gluconeogenesis; ↓ insulin; complex I stress	AMPK-mTOR suppression; reduced anabolic flux; CSC targeting; affect gut microbiome, potential radiosensitization (see footnote 1)	Epidemiologic and neoadjuvant signals + strong preclinical rationale [253,254,255,256,257,258]
Glycolysis inhibitors (2-DG) ± mitochondrial stressors	Functional ‘glucose restriction’ at the tumor cell level	Dual hit on glycolysis + OXPHOS can induce energetic crisis; synergism with fasting-like states	Additive/synergistic effects with starvation/metformin [176,259,260,261]
SGLT2 inhibitors (canagliflozin, dapagliflozin, empagliflozin)	↓ Systemic glucose exposure; facilitates nutritional ketosis in low-carb contexts	Limits substrate supply; may engage AMPK/mTOR and reduce hypoxic/HIF programs	Anticancer potential in multiple models and reviews; multi-omics HCC example [262,263]
Lipid/ketone pathway nodes (lipogenesis, ketolysis, redox)	Shifts substrate partitioning toward FAO/ketones; alters NAD+/NADH and acetyl-CoA balance	Targets anabolic lipid supply and adaptive fuel use; may modulate epigenetics and oxidative stress responses	Representative pathway landmarks include ACLY and lipid signaling networks [146,252]

Footnote 1: Metformin markedly alters gut microbiota composition, consistently increasing taxa such as Escherichia coli and the mucin-degrading bacterium *Akkermansia muciniphila*, and often enriching short-chain-fatty-acid-producing and bifidobacterial populations. These changes can improve intestinal barrier function, bile acid and SCFA metabolism, and glucose homeostasis, but may also underlie common gastrointestinal side effects. ↓ expression, secretion, or metabolic activity are decreased.

**Figure 8:**
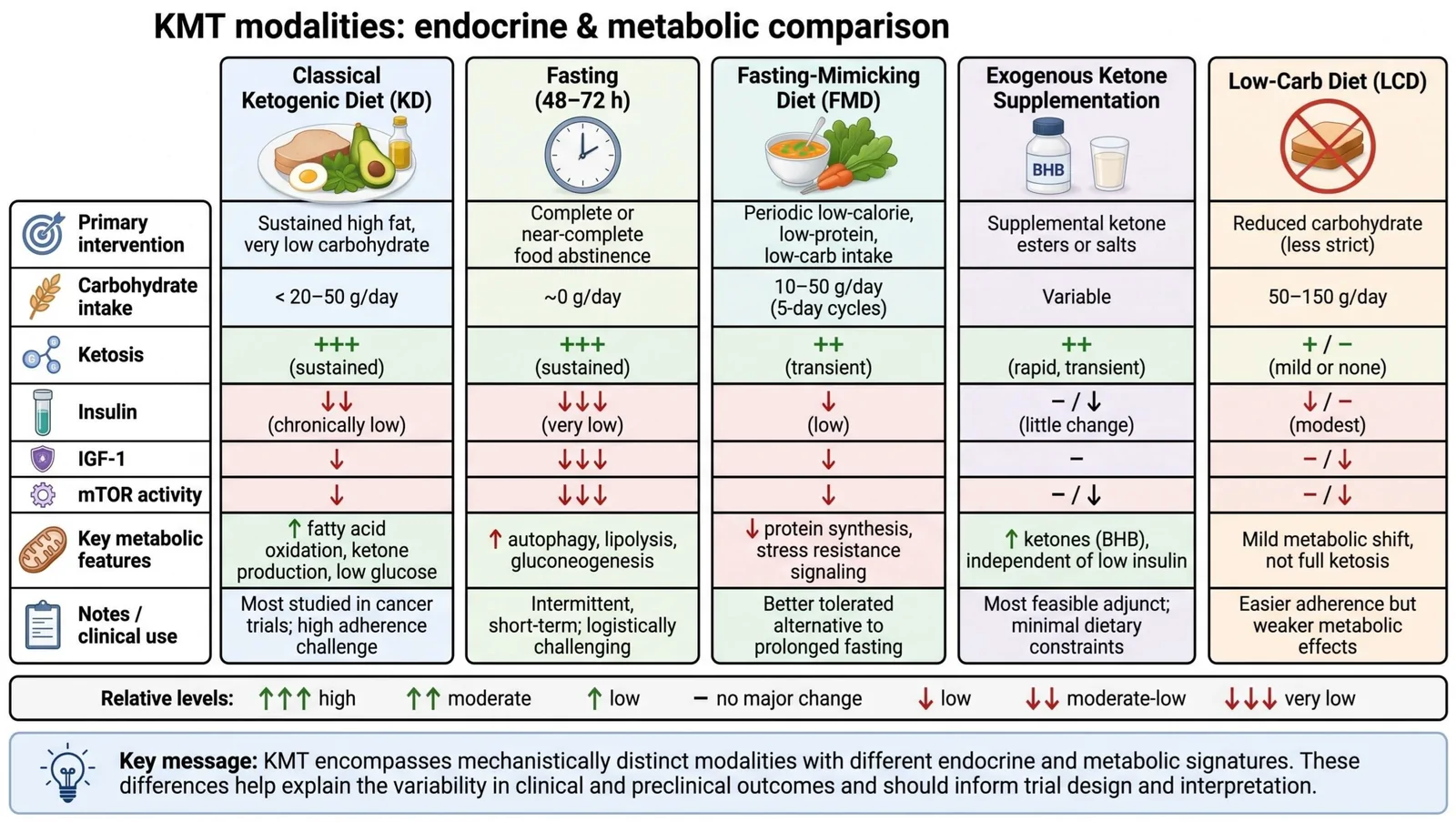
Intervention matrix: dietary and pharmacological options versus functional readouts. A matrix that exposes five candidate metabolic and pharmacological interventions (classical KD, fasting-mimicking diet, caloric restriction, exogenous ketones, and low-carb diets) against six important readouts: carbohydrate intake, state and level of ketosis, insulin levels, IGF-1 secretion, mTOR activity, and key metabolic features that can be observed and measured. Color code: red indicates that changes in nutrition have been validated to reduce levels of secretion and/or metabolic activity of the factors shown below. Green indicates that the changes in nutrition shown above increase the activity of the associated metabolic process. Light blue: no or only minor changes.

These drug classes should be viewed as complements, not replacements, for diet (Figure 8): the same metabolic plasticity that can blunt ketogenic interventions may also enable escape from single-node pharmacology, underscoring the need for tumor/host metabolic phenotyping and dynamic monitoring [246,247,264,265].

## 9. Conclusions

### 9.1. The Translational Loop

The ketogenic diet occupies a distinctive place in oncology: a long-standing metabolic therapy in neurology is increasingly explored as an adjunct to cancer treatment. Across clinical studies, KDs have generally been implementable with predictable metabolic shifts and acceptable safety profiles. However, clinical outcomes remain inconsistent, not surprisingly, because ‘KD’ is not a single intervention but a family of dietary strategies varying in fat source, macronutrient ceilings, caloric balance, and depth of ketosis achieved. Rather than weakening the premise, this variability defines the field’s central task: converting an intriguing clinical signal into predictive, mechanism-guided use. Without mechanistic grounding, we cannot identify which tumors and patients are most likely to benefit, define the biologically meaningful ‘dose,’ or rationally choose combination partners.

### 9.2. Toward Personalized Metabolic Intervention

Looking forward, the most credible vision for KD in oncology is precision use rather than broad empiricism. Metabolic interventions would be prescribed based on tumor and microenvironmental features—ketolytic capacity, immune state, stromal composition—together with patient factors (baseline insulin sensitivity, body composition, comorbidities). Diet formulations could be optimized to exploit tumor vulnerabilities while avoiding contexts in which tumors adaptively benefit. Ultimately, the ketogenic diet is best understood as a controllable perturbation of systemic and local metabolism. By closing the translational loop through rigorous, model-appropriate experimentation, the field can move from broad claims to actionable rules: when KD is likely to help, when it is unlikely to matter, and when it may carry risk. That shift from curiosity to prediction is what will transform KD from an intriguing clinical adjunct into a more rational, individual, and personalized component of precision oncology.

## Data Availability

No new data were created or analyzed in this study. Data sharing is not applicable to this article.
